# A novel fusion of genetic grey wolf optimization and kernel extreme learning machines for precise diabetic eye disease classification

**DOI:** 10.1371/journal.pone.0303094

**Published:** 2024-05-20

**Authors:** Abdul Qadir Khan, Guangmin Sun, Majdi Khalid, Azhar Imran, Anas Bilal, Muhammad Azam, Raheem Sarwar

**Affiliations:** 1 Faculty of Information Technology, Beijing University of Technology, Beijing, China; 2 Department of Computer Science and Artificial Intelligence, College of Computing, Umm Al-Qura University, Makkah, Saudi Arabia; 3 Department of Creative Technologies, Air University, Islamabad, Pakistan; 4 College of Information Science and Technology, Hainan Normal University, Haikou, China; 5 Key Laboratory of Data Science and Smart Education, Ministry of Education, Hainan Normal University, Haikou, China; 6 Department of Computer Science, Superior University, Lahore, Pakistan; 7 OTEHM, Manchester Metropolitan University, Manchester, United Kingdom; University 20 Aout 1955 skikda, Algeria, ALGERIA

## Abstract

In response to the growing number of diabetes cases worldwide, Our study addresses the escalating issue of diabetic eye disease (DED), a significant contributor to vision loss globally, through a pioneering approach. We propose a novel integration of a Genetic Grey Wolf Optimization (G-GWO) algorithm with a Fully Convolutional Encoder-Decoder Network (FCEDN), further enhanced by a Kernel Extreme Learning Machine (KELM) for refined image segmentation and disease classification. This innovative combination leverages the genetic algorithm and grey wolf optimization to boost the FCEDN’s efficiency, enabling precise detection of DED stages and differentiation among disease types. Tested across diverse datasets, including IDRiD, DR-HAGIS, and ODIR, our model showcased superior performance, achieving classification accuracies between 98.5% to 98.8%, surpassing existing methods. This advancement sets a new standard in DED detection and offers significant potential for automating fundus image analysis, reducing reliance on manual examination, and improving patient care efficiency. Our findings are crucial to enhancing diagnostic accuracy and patient outcomes in DED management.

## Introduction

Diabetes mellitus, commonly known as diabetes, is a medical condition where the body experiences high blood sugar levels due to insufficient insulin production or inadequate insulin response. This global health issue is mainly attributed to factors like a sedentary lifestyle, obesity, aging, and unhealthy dietary habits. Recent data from the International Diabetes Federation indicates a concerning increase in diabetes cases, with current figures showing 116 million people affected and projections suggesting a rise to 700 million by 2045 [1]. Diabetic eye disease (DED) encompasses many disorders, such as diabetic macular edema, diabetic retinopathy, cataracts, and Glaucoma, all of which are significant complications of diabetes. DED is a leading cause of blindness and visual impairment in the working-age population. Its symptoms, such as abnormal blood vessel growth and macular swelling, primarily affect the retina. While there are several treatment options, such as corticosteroids, laser photocoagulation, as well as anti-vascular endothelial growth factor injections, it is crucial to discover DED at an early stage to minimize vision loss since the earliest phases of the condition are often overlooked [2,3].

The increasing number of diabetes patients worldwide is straining the availability of retinal specialists, leading to delays in screening and diagnosis. Automated DED screening systems offer a solution by providing rapid, cost-effective point-of-care screening. Unlike the labor-intensive manual examination of color retinal fundus images, these automated systems can swiftly analyze images during routine screenings, facilitating early detection and treatment. Early detection and prompt treatment may prevent up to 90% of visual loss caused by DED [4]. The World Health Organization cautions that without appropriate management, the prevalence of disorders such as diabetic macular edema and diabetic retinopathy is projected to rise significantly by 2024 and 2034, respectively. Glaucoma is anticipated to become more widespread, especially among the elderly population and those with diabetes. Deploying automated DED detection devices is crucial in addressing the possible increase in visual impairment caused by diabetic eye disorders [5].

The latest advances in computer vision and artificial intelligence have profoundly impacted the development of diagnostic systems for face recognition [6], disease diagnosis [7–10], etc. Important image processing methods are the backbone of computer vision applications [11–14]. Imprecise diagnosis of Diabetic Retinopathy (DR), a more severe form of Diabetic Eye Disease (DED), may lead to significant blindness. Patients with diabetes should have their eyes checked often because of the risk of retinal damage from uncontrolled blood sugar. The delicate retina is located in the eye and converts light into neural signals sent to the brain to create visual pictures. The condition known as diabetic retinopathy (DR) causes vision impairment and retinal edema when fluids seep into the retina from damaged blood vessels.

In fundus images used for diagnosis, various structures, like the optic disc, optic cup, macula, and fovea, and potential DR lesions, such as microaneurysms, hemorrhages, and exudates, are identifiable. Symptoms like blurred vision, vision fluctuations, night vision difficulties, and vision loss in diabetic patients may indicate DR, necessitating specialist consultation. Diagnosis typically involves examining retinal fundus photographs and the patient’s diabetes history, which can be time-consuming and error-prone. Lately, computer-aided diagnosis systems, enhanced by deep learning models, have simplified the detection of DR, providing more efficient decision support. Several studies summarize these advancements. For instance, Egunsola et al. [15] conducted a comprehensive review of DR screening, examining various studies and datasets and utilizing resources like MEDLINE and Embase. Wu et al. [16] reviewed machine learning algorithms in DR screening, highlighting the emphasis on neural networks and noting the high diagnostic accuracy and the need for external validation. Bandello et al. [17] discussed the necessity of a multidisciplinary approach to early DR management.

These investigations include more than just the diagnosis of DR. Koppu et al. [18], who introduced an illness prediction model designed for intelligent robots. Lee et al. [19] conducted a study on seven DR screening systems, highlighting the need to conduct trials using real-world data. Zhang et al. [20] examined the prompt detection of microvascular irregularities in persons without diabetic retinopathy using Optical Coherence Tomography Angiography. Heydon et al. [21] successfully used machine learning to screen diabetic retinopathy (DR) for 30,000 patients using the EyeArt program. Baget-Bernaldiz et al. [22] highlighted the importance of image quality in the diagnostic process using deep learning models on the Messidor dataset. Vujosevic et al. [23] evaluated microvascular changes in patients with diabetes, with and without signs of diabetic retinopathy (DR), using optical coherence tomography angiography (OCTA). Furthermore, Moqurrab et al. [24] used artificial intelligence methodologies, namely Convolutional Neural Networks (CNNs) and Bidirectional Long Short-Term Memory (Bi-LSTM), in their investigation of clinical notes. Janakiraman et al. [25] used meta-heuristic algorithms to improve recommendation systems for patient records of persons diagnosed with diabetes and retinopathy (DR). These and other studies illustrate the growing effectiveness of hybrid deep-learning methods.

This paper presents the development of a Kernel Extreme Learning Machine (KELM) model for picture classification. The model incorporates a distinctive optimization technique, G-GWO, to effectively adjust its hyperparameters. G-GWO is a novel methodology that integrates the characteristics of the genetic algorithm with the GWO technique. Unlike conventional approaches that need the human configuration of hyperparameters, G-GWO takes inspiration from grey wolves’ social structure and hunting tactics [26–29]. Throughout its development, GWO has undergone several changes focused on improving convergence speed and minimizing the risk of being stuck in local optima [30–32].

The innovative component of our study is incorporating a genetic algorithm (GA) into GWO, resulting in the formation of G-GWO. This combination results in a more efficient starting population, enhancing the potential for better optimization outcomes. We assessed G-GWO’s performance by comparing it with other nature-inspired optimization methods using unimodal and multimodal benchmark functions. G-GWO performed better in these comparisons over several variants of KELM, GA-KELM, GWO-KELM and GGWO-KELM, algorithms. This study applied G-GWO to optimize the KELM model’s hyperparameters, leading to an enhanced classification model. The model’s efficacy was then tested on image datasets for Diabetic Macular Edema (DME), Diabetic Retinopathy (DR), and Glaucoma (GA) from the IDRiD [33], DR-HAGIS [34], and ODIR [35] datasets.

This research makes several key contributions:

- Presenting a new combination of G-GWO techniques to optimize KELM.
- The GWO algorithm is enhanced using genetic crossover and mutation operators to increase exploration efficiency and improve solution quality.
- Validating the efficacy of G-GWO by conducting comparison studies with other nature-inspired algorithms on benchmark functions.
- Implement G-GWO for the fine-tuning of KELM hyperparameters, specifically for classification tasks.
- Through simulations on DED datasets, G-GWO surpasses other optimization algorithms in accuracy and overall performance.

The subsequent sections of the paper are organized in the following manner: Section Related Mahtodologies explores the latest progress in Fully Convolutional Networks (FCNs), KELM, and the optimization of hyperparameters using techniques inspired by nature. The section proposed methodology examines the method. The results and discussion section thoroughly discusses and evaluates the data, while the conclusion section briefly summarizes the study’s conclusions.

## Literature review

Our study builds upon foundational work in automated disease classification and prediction, leveraging advancements in optimization algorithms, deep learning models, and decision support systems as detailed in recent literature [36–38]. It showcases the efficiency of DL in capturing significant information that may be missed by human techniques [39]. Out of all the models considered, the Densenet-264 model uses deep learning, and the Chimp optimization technique demonstrates exceptional efficiency. It achieved an accuracy rate of 99.73% on the Messidor dataset [40]. The DRNet model was further modified for classification by using a Support Vector Machine (SVM) [41]. A multi-feature fusion-based Directed Acyclic Graph (DAG) network was suggested and evaluated for the classification of Diabetic Retinopathy (DR) lesions using a local dataset and DIARETDB1. The network achieved 98.70% and 98.50% accuracy on the respective datasets [42]. Feature extraction was performed using the firefly optimization (FFO) approach, with optimization carried out using the improved grey wolf optimization (iGWO) method [43].

The VGGNet architecture was used to extract high-level features from fundus photos, using transfer learning to enhance the precision of image classification [44]. The Faster-RCNN algorithm with DenseNet-65 was used to determine the position and categorization of multiclass DR lesions[45]. The research used a unique technique that included extracting characteristics from altered deep networks, including VGG19, ResNet101, and InceptionV3. Subsequently, these characteristics underwent four filter-based feature selection approaches, including MRMR, ReliefF, and F-test. Ultimately, the categorization process was executed with an SVM classifier [46]. The DFTSA-Net model, which employs four pre-trained networks (GoogLeNet, SqueezeNet, ResNet-50, Inception-v3) as feature extractors, was developed to detect DR lesions [47]. The ConvNet model contributed notably by attaining a 97.41% accuracy in accurately recognizing DR lesions on the APTOS 2019 dataset [48–51]. The Faster R-CNN algorithm was used to extract salient features from fundus images. The Softmax algorithm was used to categorize the lesions of diabetic retinopathy (DR). The accuracy of this approach was assessed using the DIARETDB1 and Messidor datasets, resulting in an impressive accuracy of 95% [52]. A CNN model was introduced, which included three pre-trained models (VGG-16, SqueezeNet, AlexNet) as primary classifiers to detect DR lesions. The Messidor dataset was used to assess the model, which achieved an accuracy of 98.15% [53]. An alternative method used five deep convolutional neural network (CNN) models to detect lesions associated with diabetic retinopathy (DR) [54]. The AlexNet architecture was used as a feature extractor, and the retrieved features were then diminished via the utilization of Principal Component Analysis (PCA) and Bag-of-Words (BoW) approaches. The decreased characteristics were ultimately categorized using a Support Vector Machine (SVM) [55].

An artificial neural network (ANN) model using the AlexNet architecture extracted features from retinal pictures. These features were then subjected to feature selection using Principal Component Analysis (PCA), Linear Discriminant Analysis (LDA), and Support Vector Machine (SVM) classification. The performance of this model was evaluated on the Kaggle dataset, achieving an accuracy of 97.93% [56]. In addition, a Residual network was used to extract profound characteristics, which were then employed for classification using a decision tree model to identify multiclass DR lesions [57]. This study emphasizes the adaptability and efficacy of deep learning methods in medical imaging, specifically in identifying and categorizing diabetic retinopathy (DR). Their research included attentional processes into the ResNet architecture, using the EyePACS dataset to classify diabetic retinopathy (DR). Their methodology consisted of augmenting the dataset using image processing methods to enhance contrast. The study highlighted that the attention mechanisms led to more effective feature extraction, resulting in an accuracy of 91.3% and a kappa value of 89.3% [58]. [39] Performed an extensive analysis, including research that used machine learning and deep learning algorithms to identify diabetic retinopathy. Their analysis emphasized the difficulties in identifying DR and proposed that deep learning models exhibit superior efficacy compared to conventional machine learning techniques. The study also found that using deep learning models for feature extraction might significantly improve performance. A total of 40 research studies were analyzed, consisting of 11 supervised, three self-supervised, and four transformer works that specifically addressed the detection, classification, and segmentation of DR [59]. Their findings indicated recent successes in classification studies, though challenges remain in identifying affected lesions. The reviews suggested the need for automatic diagnostic systems with full clinical potential.

This study aims to introduce a novel approach that enhances the development of automatic identifier systems while meeting specific requirements. Additionally, previous research has recognized substantial improvement in detecting and classifying conditions like DR (Diabetic Retinopathy), DME (Diabetic Macular Edema), and Glaucoma through image processing. Our method aims to capitalize on this critical aspect. A summary of related work in automated disease classification and prediction is presented in Table 1.

**Table 1:** Summary of related work.

Reference	Dataset	Features Extraction	Classification	Results
[36–38]	Not specified	Optimization algorithms, deep learning	Decision support systems	Not specified
[39]	Messidor	Deep learning (Densenet-264)	Not specified	99.73% accuracy
[40]	Local, DIARETDB1	Multi-feature fusion, DAG network	SVM	98.70%, 98.50%
[41]	Not specified	Firefly optimization (FFO)	Grey wolf optimization (iGWO)	Not specified
[44]	Not specified	VGGNet, transfer learning	Not specified	Not specified
[45]	Not specified	Faster-RCNN, DenseNet-65	Not specified	Not specified
[46]	Not specified	VGG19, ResNet101, InceptionV3	SVM	Not specified
[47]	APTOS 2019	GoogLeNet, SqueezeNet, ResNet-50, Inception-v3	ConvNet	97.41% accuracy
[48–51]	DIARETDB1, Messidor	Faster R-CNN	Softmax	95% accuracy
[52]	Messidor	VGG-16, SqueezeNet, AlexNet	CNN	98.15% accuracy
[53]	Not specified	CNN models	SVM	Not specified
[54]	Not specified	AlexNet	PCA, BoW, SVM	Not specified
[55]	Kaggle	AlexNet	PCA, LDA, SVM	97.93% accuracy
[56]	Not specified	Residual network	Decision tree	Not specified
[57]	EyePACS	ResNet with attentional processes	Image processing enhancements	91.3% accuracy, kappa value of 89.3%

## Related methodologies

This section provides an overview of the basic principles behind the G-GWO optimization method and its connection to the architecture and hyperparameters of KELM and FCEDN.

### Fully Convolution Encoder-Decoder Network (FCEDN)

Convolutional Neural Networks (CNNs) are well respected in computer vision due to their strong skills in extracting features, making predictions, and performing classification tasks [60]. However, their application to image segmentation can be challenging. Standard CNNs, initially designed for image classification, tend to underperform in segmentation tasks as their fully connected layers overlook spatial data, providing only a single class probability instead of the necessary pixel-level classification for semantic segmentation.FCNs have been devised as a solution to this issue, substituting the fully connected layers within CNNs with convolutional as well as deconvolutional layers. Due to the removal of thoroughly combined layers, FCNs are advantageous for their efficiency and time-saving characteristics [61]. In semantic segmentation using FCNs, two primary methods are employed:

#### Fundamental FCN architecture

This method constructs the FCN with convolutional (Conv), Rectified Linear Unit (ReLU), pooling (PL), and upsampling (UP) layers [61]. Conv and PL are responsible for downsampling image features, while the UP layer handles the final upsampling. However, this approach can sometimes lead to suboptimal performance due to the absence of a trainable UP layer, which might result in the loss of spatial information [60].

#### Encoder-Decoder approach

The Encoder-Decoder Approach is a sophisticated technique that has two components: an encoder, which is made up of CNN-like layers, and a decoder, which utilizes transposed convolution (TC) and UP layers to increase the resolution of feature maps[62]. Integrating trainable parameters into the upsampling layers dramatically improves the accuracy of semantic segmentation.

**Fig 1:**
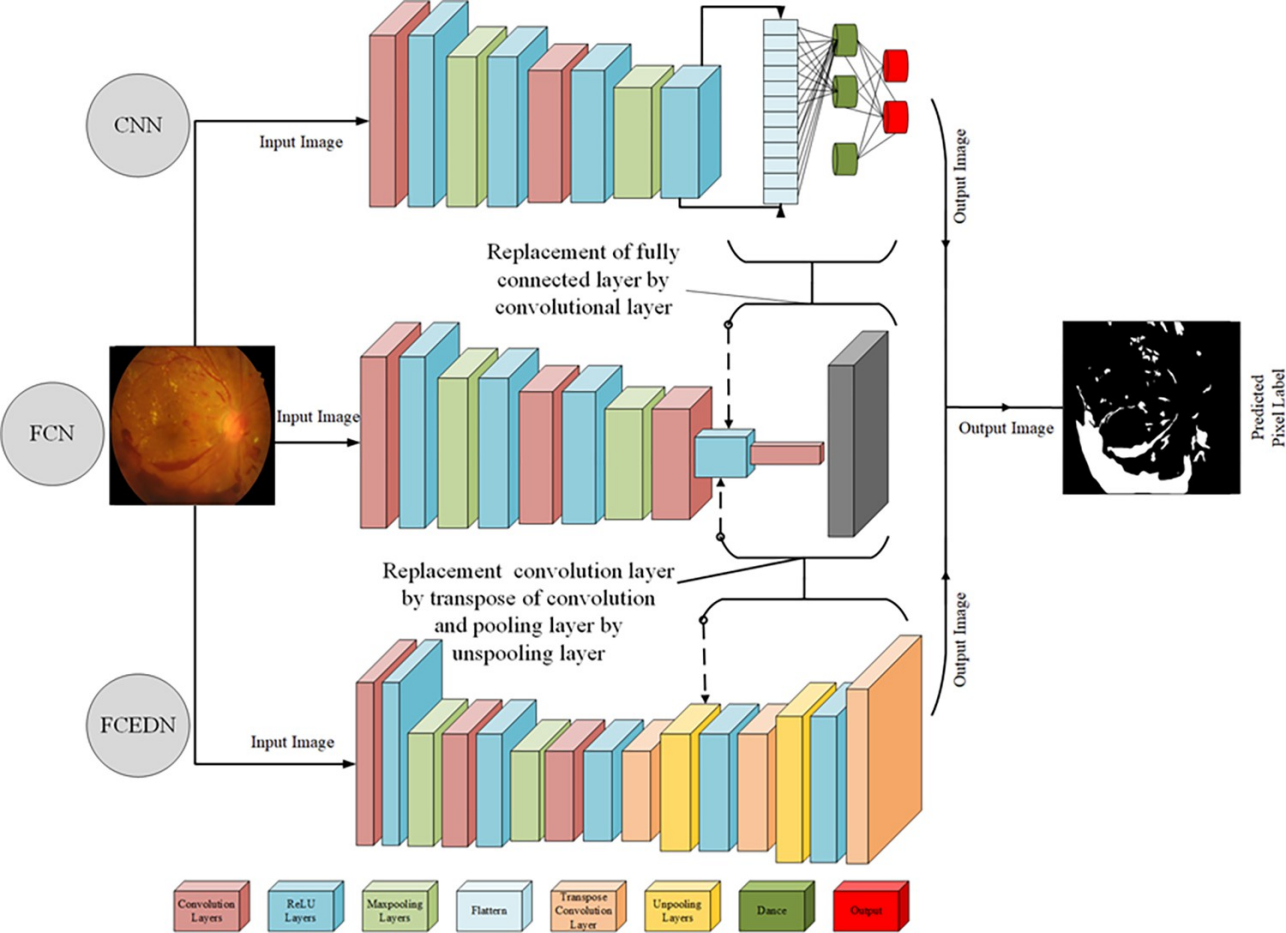
CNN, FCN, and FCEDN architecture.

This study introduces a Fully Convolutional Encoder-Decoder Network (FCEDN) to enhance pixel-level segmentation. The FCEDN utilizes convolutional, dropout, and max pooling (MP) layers to extract and downsample information via the encoder. The decoder, which includes trainable TC (transposed convolution), UP (upsampling), and dropout layers, systematically performs upsampling (US) on the encoded output. This process culminates in an output layer that aligns with the input image’s ground-truth dimensions. Compared to traditional FCNs with a non-trainable layer for the US, our FCEDN architecture, featuring a dual trainable encoder/decoder design, demonstrates superior segmentation performance. Fig 1 in our study visually compares the architectures of FCEDN, CNN, and FCN, highlighting the advancements and effectiveness of our proposed FCEDN model.

### Kernel Extreme Learning Machine (KELM)

The traditional backpropagation (BP) learning algorithm, known as a stochastic gradient least mean square algorithm, encounters challenges with noise interference in training samples. This noise significantly influences each gradient iteration in BP. The batch method is often employed to mitigate this, averaging the gradients over multiple samples to estimate the actual gradient. However, this approach becomes computationally intensive with many training samples and may overlook the unique characteristics of individual samples, thereby diminishing the learning sensitivity [63].

Against this backdrop, exploring Extreme Learning Machines (ELM) presents a compelling advancement. As recent studies [64–67] showcased, ELM offers an innovative approach by enhancing detection and recognition capabilities across various domains without the computational and sensitivity drawbacks associated with BP. This evolution from traditional BP to ELM underlines a significant leap in addressing and overcoming the challenges posed by noise in training samples, ushering in a new era of efficiency and precision in machine learning applications.

To address these limitations, the Kernel Extreme Learning Machine (KELM) is an enhancement of the original Extreme Learning Machine (ELM) algorithm, which incorporates kernel functions [63]. The ELM guarantees excellent generalization performance in networks and dramatically enhances the learning pace of forward neural networks. It successfully addresses the limitations of training techniques based on gradient descent, as shown in BP neural networks, which are susceptible to becoming stuck in local optima and need many iterations.

KELM distinguishes itself by using several of the benefits of the ELM method and integrating kernel functions. These kernel functions facilitate the transformation of linearly non-separable patterns into a feature space with larger dimensions, resulting in linear separability and improved accuracy. KELM’s robustness makes it an excellent option for intricate learning problems.

ELM is a training technique for single-layer feedforward neural networks (SLFNs). The SLFNs model can be described as follows [64]:

f(x)−h(x)β=Hβ (1)

The model emphasizes the fundamental structure of ELM and its efficacy in managing diverse learning situations, which is further enhanced by KELM’s sophisticated kernel-based approach.

In the context of the Extreme Learning Machine (ELM) algorithm, the sample being analyzed is denoted as ’*x*.’ The result of the neural network for this example represented as ’*f*(*x*;),’ usually corresponds to a vector of class labels in classification tasks. The feature mapping of the hidden layer is denoted as ’*h*(*x*)’ or ’*H*,’ which converts the input data into a feature space. The weights connecting the hidden layer to the output layer are represented by the symbol ’*β*.’

The ELM algorithm utilizes this framework to handle the incoming data using the neural network. The hidden layer feature mapping ’H’ is essential for converting the input ’x’ into a significant representation that the output layer can efficiently utilize. The output layer utilizes the weights ’*β*’ to interpret these characteristics and provide the ultimate output ’*f*(*x*),’ which is used for classification or other prediction endeavors. The efficiency and success of the ELM algorithm depend on its organized approach to data processing inside the neural network.


$$\beta =H^{T}{\left(HH^{T}+\frac{I}{C}\right)}^{-1}T$$ (2)

In the framework for training neural networks, ’T’ denotes a matrix that holds the class flag vectors of the training samples. These vectors are crucial for determining the groups or classes each sample belongs to. Furthermore, the symbol ’*I*’ represents the identity matrix, an essential component in matrix operations. The identity matrix is distinguished by having ones along the diagonal and zeros at all other positions. The word ’C’ represents the regularization parameter, which plays a critical role in balancing the accuracy of fitting the training data with the simplicity of the model parameters to prevent overfitting. When the mapping ’*h*(*x*)’ of the hidden layer is not immediately known or visible, a kernel matrix becomes relevant, especially in the context of Kernel Extreme Learning Machines (KELM). The KELM kernel matrix is specifically designed to address such problems adequately. The matrix is crucial in KELM since it allows for calculating the required transformations without needing explicit knowledge of ’*h*(*x*).’ The kernel matrix is formulated as follows [65]:

$$\Omega =HH^{T}:\Omega_{i,j}-h(x_{i})=K(x_{i},x_{j})$$ (3)

The kernel matrix methodology enables KELM to function well even when direct access to the hidden layer feature map is impossible. This strategy successfully harnesses the capabilities of kernel methods to analyze and categorize the training data. According to (2) and (3), (1) can be transformed as follows:

$$f(x)-H\beta ={HH^{T}(HH^{T}+\frac{I}{C})}^{-1}T$$

$${\left[\begin{array}{c} K(x,x_{1})\\ \vdots \\ K(x,x_{N}) \end{array}\right]}^{T}(\Omega {+\frac{I}{C})}^{-1}T$$ (4)

The Radial Basis Function (RBF), sometimes called the Gaussian kernel function [66], may be mathematically described when used as the kernel function in computational models. The RBF kernel is renowned for its efficacy in diverse machine-learning methods, especially when involving nonlinear data. Its capability to convert data into a higher-dimensional space distinguishes it, enabling more efficient classification or regression tasks.

The Gaussian kernel function, a commonly used option within the RBF kernel family, is characterized by its precise mathematical formulation. The RBF kernel’s functionality is encapsulated by this equation, allowing it to effectively handle intricate patterns in data by quantifying the similarity between various locations in the feature space. The precise understanding of this kernel function is crucial for its utilization in different algorithms, guaranteeing the most efficient execution across a wide range of computing activities.


$$K(x,x_{1})=exp(-\frac{{\left\|x-y\right\|}^{2}}{2\gamma^{2}})$$ (5)

The system’s performance is greatly influenced by two critical parameters: the regularization value, indicated as ’C,’ and the kernel function parameter, represented by ’gamma.’ Precisely adjusting these parameters is crucial for maximizing the classifier’s efficiency. The regularization parameter ’C’ is essential for managing the trade-off between the complexity of the model and its capacity to generalize to unseen data. Conversely, the parameter ’*γ*’ of the kernel function governs the characteristics of the kernel function, which is crucial for the classifier’s capacity to deal with non-linear associations in the data.

**Fig 2:**
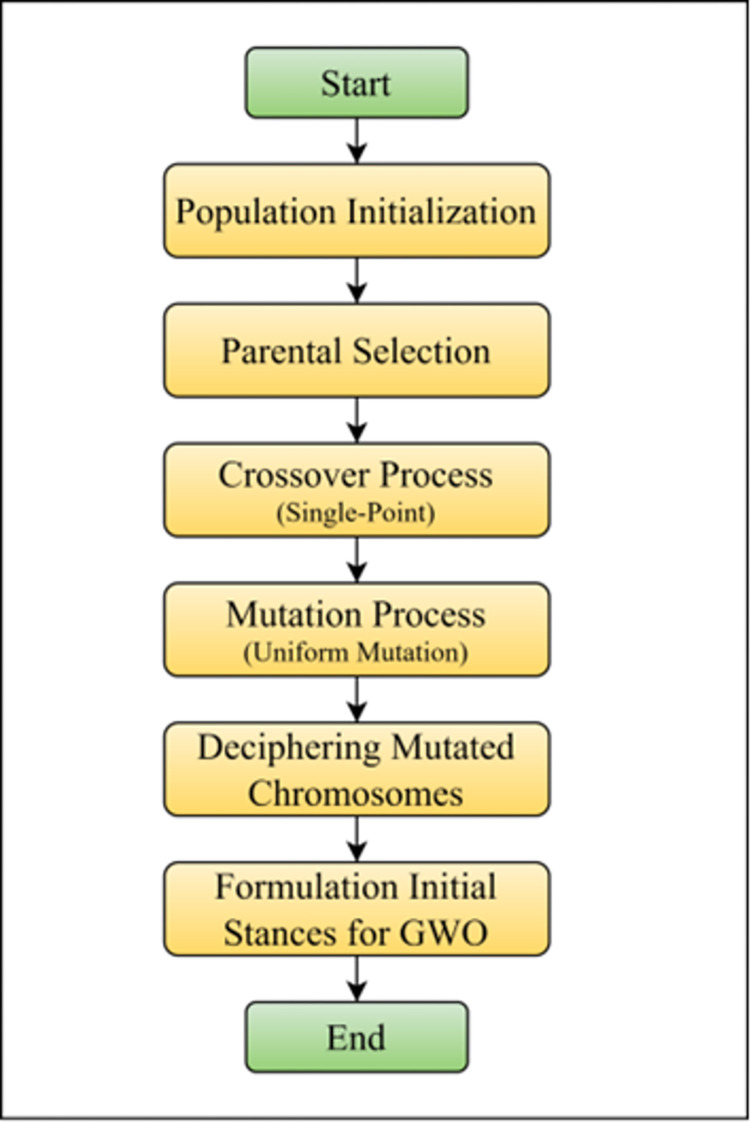
KELM configuration.

The precise arrangement of both ’C’ and ’*γ*’ is crucial since it directly affects the KELM’s capacity to categorize data appropriately. The parameters must be carefully fine-tuned to guarantee that the classifier operates at its best, accurately discriminating between various classes and adjusting to the unique properties of the dataset it is applied to. Fig 2 offers a visual representation of the KELM’s workings.

### Genetic Algorithm (GA)

The Genetic Algorithm (GA), first proposed by Holland [67], draws inspiration from the ideas of Darwinian natural selection and genetic processes found in biological systems. Genetic algorithm (GA) is an adaptive optimization technique that uses a collection of potential solutions called chromosomes. The chromosomes consist of many genes, usually represented by binary values of 0 or 1. In this work, we use a Genetic Algorithm (GA) to produce the starting locations for the GWO algorithm. The generation of these initial placements using Genetic Algorithms (GA) entails a series of crucial stages:

- Population initialization begins by randomly generating chromosomes, forming the starting population.
- Parental Selection: The roulette wheel selection technique picks parent chromosomes. This method emulates a roulette wheel, where the likelihood of choosing a chromosome is proportional to its fitness.
- Crossover Process: Specifically, we employ a single-point crossover operation, where the production of child chromosomes requires the exchange of genetic information between parent chromosomes at a randomly selected location across their length. This technique ensures a mix of parental traits with a chance of producing more viable offspring.
- Mutation Process: We utilize a uniform mutation strategy to provide genetic variety and prevent early convergence. This approach involves randomly altering gene values with a fixed probability, introducing new features in the child’s chromosomes, and enhancing the genetic diversity within the population.
- Deciphering Mutated Chromosomes: The modified chromosomes are then decoded to determine the original positions of the population within the solution space.

**Fig 3:**
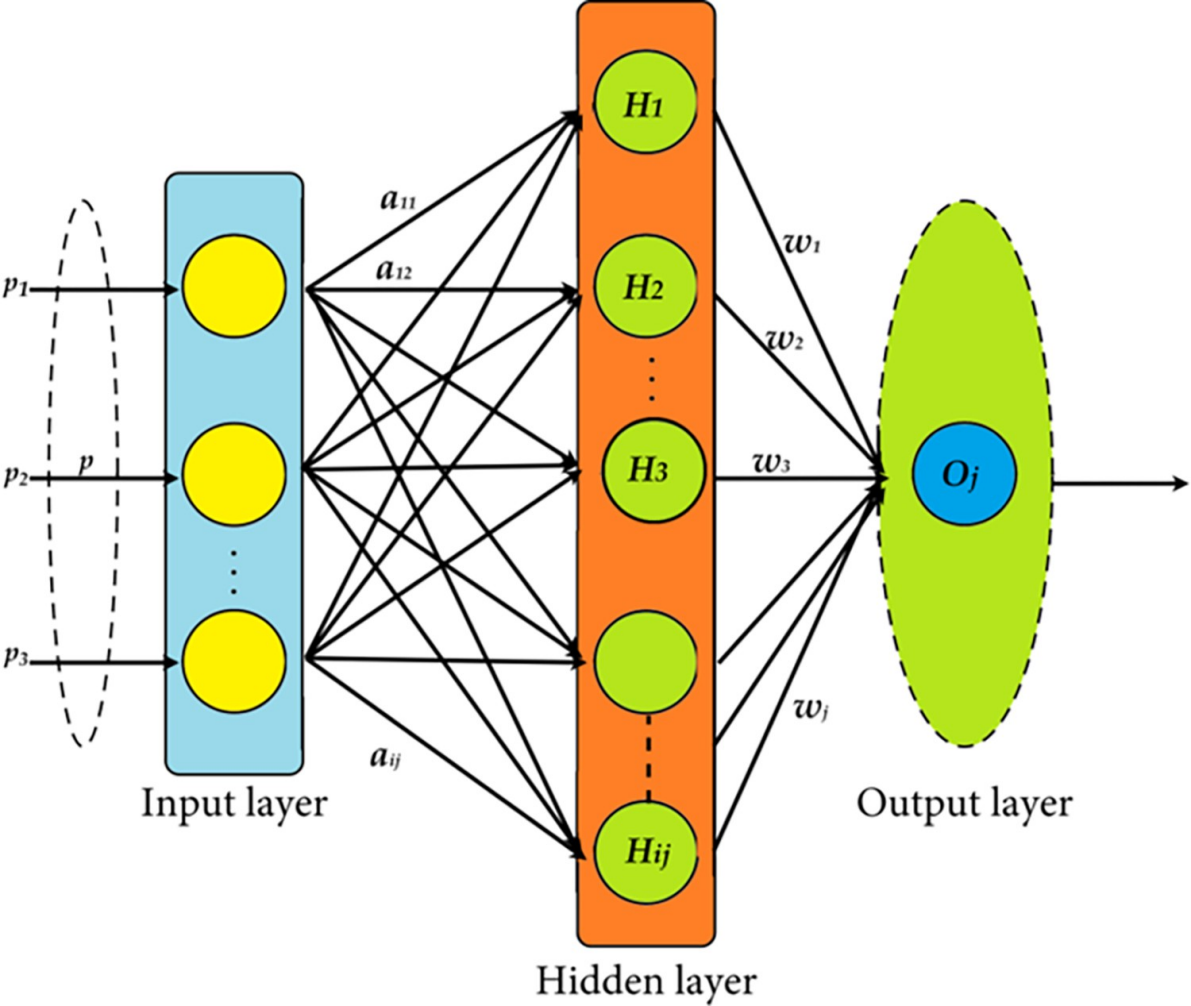
Genetic algorithm flowchart.

Formulating Initial Stances for GWO: Incorporating genetic algorithms (GA) into the process is essential. It establishes the groundwork for the following optimization stages, facilitating a more streamlined and successful pursuit of optimum solutions. For a detailed overview of the Genetic Algorithm process utilized in this study, please refer to the flowchart presented in Fig 3.

### Genetic-Grey Wolf Optimization (G-GWO) algorithm

The GWO algorithm [26] is a metaheuristic algorithm that draws inspiration from grey wolves’ social structure and hunting behavior (GWs). This algorithm simulates the intricate collective behavior of wolves during the hunt, with a specific emphasis on three main phases: surrounding the prey, pursuing it, and launching an assault.

The social organization of the wolf pack in GWO is mathematically simulated, where the ranking is established based on the optimal solutions. The alpha wolf (α) is the ideal solution within the pack, symbolizing its position as the leader. Beta (β) and delta (δ) wolves represent the second and third perfect solutions. The alpha depends on these wolves to assist in both decision-making and hunting. The omega (ω) wolves represent the remaining members of the pack who follow after the alpha, beta, and delta wolves.

The GWO algorithm utilizes a mathematical equation, Eq 6, to replicate the surrounding behavior of grey wolves when hunting. This equation represents the wolves’ tactic of encircling their prey, a vital element of their hunting style. The encircling mechanism in GWO is essential as it serves as the foundation of the algorithm’s search and optimization process, directing the movement of the wolf pack (solutions) toward the prey (ideal solution). The correct articulation of this behavior is crucial in determining the algorithm’s efficacy in exploring and utilizing the solution space.


$$\vec{Y}=|\vec{Q}\times {\vec{M}}_{p}(t)-{\vec{M}}_{w}|,{\vec{M}}_{w}(t+1)={\vec{M}}_{p}(t)-\vec{N}\times \vec{Y}|$$ (6)

The GWO method utilizes distinct symbols and vectors to represent different aspects and activities. These depictions are essential for comprehending the mathematical operations of the algorithm. Let’s analyze their significance within the framework of GWO:

Current Iteration (*t*): This symbol denotes the present stage or cycle in the optimization process.

In the GWO algorithm, the prey represents the goal or the ideal solution the grey wolves (GWs) want to achieve.

Grey Wolf (*w*): A grey wolf symbolizes a possible solution inside the algorithm.

The coefficient vectors ($\vec{N}$ and $\vec{Q}$) are essential for directing the movement of the grey wolves towards their prey. The dynamic changes with each Iteration influence the wolves’ placements in the search space.

The prey’s location vector (${\vec{M}}_{p}$) represents the precise position of the prey inside the search space. It serves as a guide for the wolves, leading them towards the most optimum option.

The Location Vector of the Grey Wolf (${\vec{M}}_{w}$) provides the precise location of a grey wolf, which is a possible solution inside the search space.

Determining the coefficient vectors $\vec{N}$ and $\vec{Q}$ This is a crucial step in the method, as they enables the mathematical representation of the wolves’ surroundings and hunting behavior. The computations are given in Eqs 7 and 8 correspondingly. The equations play a crucial role in the GWO algorithm, as they dictate the behavior and effectiveness of the grey wolves in their pursuit of the prey, ultimately leading to the discovery of the best possible solution. The precise formulation of these vectors and their interaction is crucial for the effectiveness of the GWO method in addressing optimization issues.


$$\vec{N}=2\vec{n}\times {\vec{l}}_{1}-\vec{n}$$ (7)

$$\vec{Q}=2{\vec{l}}_{2}$$ (8)

The GWO method utilizes many vectors to direct the simulated hunting behavior of grey wolves toward an ideal solution. A vector, represented as $\vec{n}$, is crucial since it progressively decreases linearly from 2 to 0 over the repetitions. This slow drop is intended to replicate the gradual approach of wolves toward their prey as the hunt advances.

In addition, the algorithm utilizes two randomly chosen vectors, ${\vec{l}}_{1}$ and ${\vec{l}}_{2}$, from the range [0,1]. These vectors enhance the stochasticity of the wolves’ search, adding a random element that aids in a more comprehensive search space exploration.

Eqs 6–8 in the GWO framework are designed to mimic the tactical hunting maneuvers of grey wolves. The mathematical representations alpha (α), beta (β), delta (δ), and omega (ω) play a crucial role in directing the simulated wolves toward the prey, which represents the best possible solution in the search space.

During each iteration of the program, the positions of the wolves are updated according to these equations. The alpha wolf, symbolizing the most optimal present resolution, mostly leads the group. The beta and delta wolves, representing the second and third most optimal alternatives, also contribute to the direction of the hunt. The other members of the pack, known as the omega wolves, adhere to the guidance of these leaders, modifying their places according to Eqs 9 to 15 [22].

The hierarchical structure and the accompanying equations allow the GWO algorithm to navigate and utilize the search space efficiently. The algorithm can achieve convergence to the most promising solutions by iteratively updating the positions of the wolves, thus emulating the efficient and cooperative hunting behavior of grey wolves in their natural habitat.


$${\vec{Y}}_{\alpha }={\vec{Q}}_{1}\times ({\vec{M}}_{\alpha }-\vec{M})$$ (9)

$${\vec{Y}}_{\beta }={\vec{Q}}_{2}\times ({\vec{M}}_{\beta }-\vec{M})$$ (10)

$${\vec{Y}}_{\delta }={\vec{Q}}_{3}\times ({\vec{M}}_{\delta }-\vec{M})$$ (11)

$${\vec{M}}_{1}={\vec{M}}_{\alpha }-({\vec{N}}_{1}\times {\vec{Y}}_{\alpha })$$ (12)

$${\vec{M}}_{2}={\vec{M}}_{\beta }-({\vec{N}}_{2}\times {\vec{Y}}_{\beta })$$ (13)

$${\vec{M}}_{3}={\vec{M}}_{\delta }-({\vec{N}}_{3}\times {\vec{Y}}_{\delta })$$ (14)

$$\vec{M}(t+1)=\frac{{\vec{M}}_{1}+{\vec{M}}_{2}+{\vec{M}}_{3}}{3}$$ (15)

In the original GWO method, randomly creating the initial population of wolf swarms may sometimes lead to a deficiency of variation within the group as they explore the search space. Studies on swarm intelligence optimization algorithms have constantly emphasized the significance of the starting population’s quality. This attribute is crucial in attaining worldwide harmonization and acquiring top-notch solutions.

A novel technique called G-GWO has been devised to improve the efficiency of the GWO algorithm. This novel technique incorporates the concepts of Genetic Algorithms (GA) to generate the first wolf swarm. G-GWO aims to provide a varied and appropriate starting population to enhance the optimization process.

Utilizing the G-GWO confers a significant strategic benefit. The algorithm’s exploring capabilities may be considerably enhanced by introducing a more organized and diverse starting place for the wolf swarm. This approach considers a broader array of possible solutions from the beginning, improving the probability of the algorithm reaching the best possible outcome. The G-GWO technique signifies a significant improvement in enhancing the efficiency of the GWO algorithm.

## Proposed methodology

**Fig 4:**
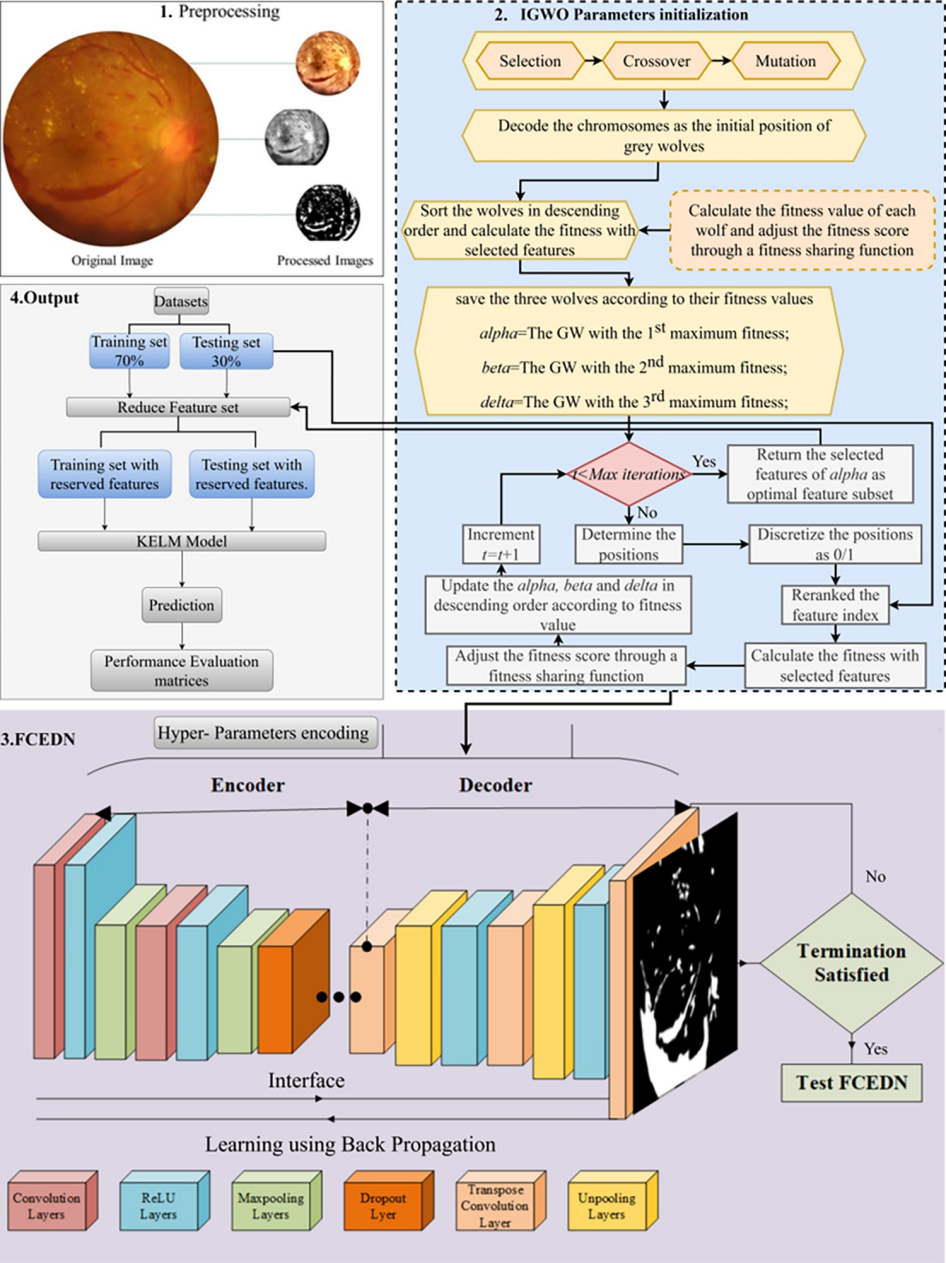
Proposed methodology.

The proposed model consists of four fundamental processes, as seen in Fig 4. The approach comprises many stages: image pre-processing, using the G-GWO algorithm to select hyperparameters, constructing and training a KELM model with the chosen hyperparameters, and assessing the model’s performance [68].

To ensure the highest efficiency and accuracy in our model, we meticulously selected the Genetic Grey Wolf Optimization (G-GWO) and Kernel Extreme Learning Machine (KELM) algorithms for their specific strengths in handling the challenges presented by Diabetic Eye Disease (DED) classification and segmentation.

The G-GWO was chosen due to its robust optimization capabilities, particularly in complex search spaces. This algorithm effectively combines the explorative power of Genetic Algorithms (GA) with the exploitative strength of Grey Wolf Optimization (GWO), making it adept at finding global optima while avoiding local optima traps. Its application in our methodology is crucial for optimizing the hyperparameters of the KELM model, ensuring that we achieve the best possible performance in terms of accuracy and computational efficiency. The G-GWO’s ability to dynamically adapt and refine search strategies is particularly beneficial for processing the high-dimensional data associated with DED images, where conventional optimization methods often fall short.KELM was selected for its rapid training and exceptional generalization performance. Unlike traditional neural networks, KELM requires no iterative tuning, making it significantly faster and more efficient, especially when dealing with large datasets. Its kernel function enhances the model’s ability to handle non-linear data, providing superior classification accuracy in medical image analysis. In the context of DED, where timely and accurate diagnosis is crucial, KELM’s fast processing and high predictive accuracy make it an ideal choice for classification and segmentation tasks.

Furthermore, the proposed methodology, integrating Genetic Grey Wolf Optimization (G-GWO) with Kernel Extreme Learning Machine (KELM), represents a significant advancement in Diabetic Eye Disease (DED) classification and segmentation. The novelty of our approach lies in its efficient optimization process and rapid, accurate predictive modeling, which is tailored for large-scale medical image analysis.

In [69], IoT and 5G with AI for real-time VTDR diagnosis, employing a multi-model AI-driven framework that includes a hybrid CNN-SVD for feature extraction and classification through SVM-RBF, DT, and KNN. While demonstrating high accuracy, this approach requires complex system integration and extensive data processing. In contrast, our methodology simplifies the computational process without compromising accuracy or efficiency. By leveraging G-GWO, we enhance hyperparameter optimization in KELM, achieving streamlined and scalable model training suitable for rapid deployment in various healthcare settings. This is especially beneficial for regions with limited access to advanced technological infrastructure.

Moreover, [70] enhances VTDR detection through a Hierarchical Block Attention (HBA) and HBA-U-Net architecture, emphasizing detailed pixel analysis for improved image segmentation. Although effective in achieving high-performance metrics, the methodology necessitates substantial computational resources. Our approach distinguishes itself by optimizing the entire process through the synergy of G-GWO and KELM, facilitating enhanced performance with reduced computational demand. This efficiency enables more effective processing of extensive DED image datasets, making it a more feasible and robust solution for widespread clinical application.

The proposed methodology showcases improved efficiency and scalability in DED image analysis and underscores our commitment to advancing medical diagnostics through AI. By optimizing hyperparameters and model architecture with G-GWO and KELM, we provide a novel, efficient, and scalable solution that outperforms existing methods regarding computational efficiency and practical applicability, marking a clear advancement in medical image analysis.

### Experimental design and datasets description

Extensive experiments were done to evaluate the efficacy of G-GWO in optimizing the hyperparameters of the Kernel Extreme Learning Machine (KELM).

This study enhances diabetic eye disease classification and suggests possibilities for improved healthcare integration, which could boost early detection and patient outcomes. Since the datasets IDRiD[33], DR-HAGIS[34], and ODIR [35] were used in this study and are publicly available, there is no need for an ethics statement. The datasets played a crucial role in evaluating the algorithm’s ability to extract features based on super pixels, classify them, and provide accurate reference data.

The studies used a comprehensive set of tools and libraries, such as MATLAB, Python, Keras, Scikit-learn, and OpenCV, to streamline data processing and analysis. The experimental setup for these tests used Google Colab Pro, which had a high-performance GPU, an Intel-Core i7 8th generation CPU, and 32 GB of RAM. This configuration guaranteed the effective management and analysis of the datasets.

The IDRiD, DR-HAGIS, and ODIR datasets are comprised of various image resolutions, such as 4288 × 2848, 4752 × 3168, 3456 × 2304, 3126 × 2136, 2896 × 1944, and 2816 × 1880, offering a diverse range of image qualities and sizes for analysis. The IDRiD dataset has precisely 516 RGB pictures designed for classification applications. The DR-HAGIS dataset has 30 RGB photographs, whereas the ODIR dataset contains 362 RGB photos. The ODIR dataset has 177 photographs depicting Glaucoma, 49 pictures illustrating diabetic retinopathy (DR), and 136 images showcasing diabetic macular edema (DME). This dataset provides a comprehensive collection of many diagnostic scenarios.

**Fig 5:**
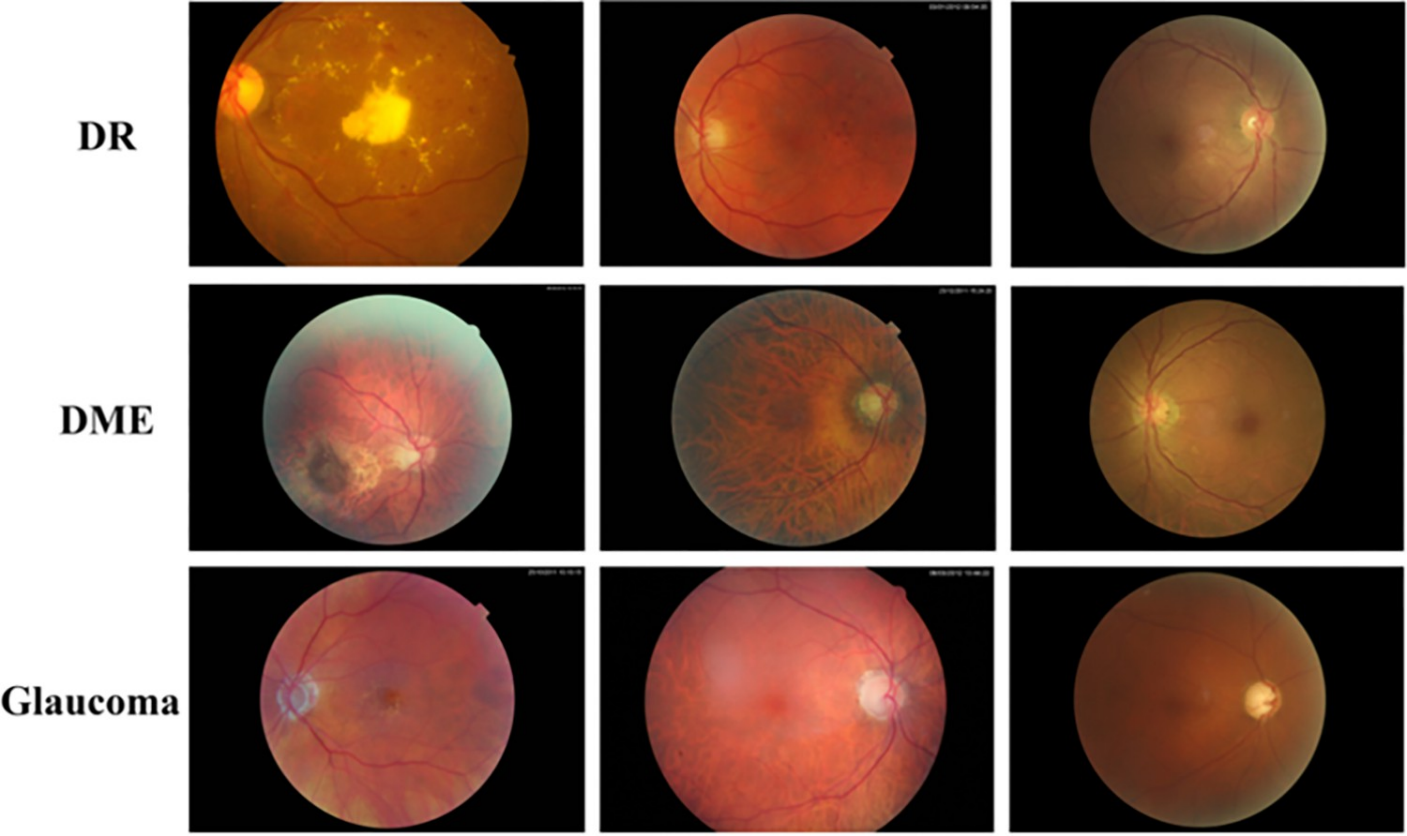
Sample images from the datasets.

To get a comprehensive analysis of the datasets used and to see example photographs, readers are directed to Table 2 and Fig 5 in the paper. These materials provide a more comprehensive understanding of the dataset’s properties and the visual context of the pictures used in the trials. They emphasize the strength and variety of the data used to evaluate the G-GWO’s effectiveness in hyperparameter optimization for KELM.

**Table 2:** A Detailed description of the dataset.

Fundus image Datasets	DR	DME	Glaucoma	Total Images
IDRiD [33]	516		0	516
DR-HAGIS [34]	10	10	10	30
ODIR [35]	1131	171	207	1509
Total Fundus Images	-	-	-	2055

The IDRiD [33] is a distinct collection exclusively created for India, including 516 retinal fundus images. The photos were taken at the eye clinic in Nanded, Maharashtra, using a Kowa VX-10α fundus camera. The dataset specifically targets the macula and provides a wide field of view of 50 degrees, enabling comprehensive examinations of diabetic retinopathy and normal retinal structures. Each image is meticulously labeled with accurate information at the pixel level. The grading methodology enhances the dataset by giving values ranging from 0 to 4 for diabetic retinopathy and 0 to 3 for diabetic macular edema. The grades of IDRiD adhere to internationally recognized clinical criteria, making it an essential tool for developing algorithms designed to detect and evaluate diabetic retinopathy in India promptly.

The DR-HAGIS[34] comprises retinal fundus pictures acquired from the diabetic retinopathy screening program in the United Kingdom. The collection consists of 39 high-resolution, color fundus images. The screening procedure employs a variety of fundus and digital cameras obtained from various service providers, resulting in many image resolutions and sizes. The database contains images of diabetic retinopathy, along with four other subsets of comorbidities: age-related macular degeneration, hypertension, and Glaucoma. The primary objective of the DR-HAGIS database is to faithfully depict the vast array of photographs that experts assess during screenings.

The Ocular Disease Recognition (ODIR) [35]consists of a comprehensive collection of retinal images captured using fundus cameras. The primary purpose of this dataset, which is accessible to the public, is to further research in identifying ocular illnesses. It enables the development and evaluation of algorithms for detecting and classifying eye-related disorders. The dataset has a diverse collection of photographs depicting persons with various ocular illnesses and those in good condition. This enables a thorough comparison. The dataset is annotated for conditions such as diabetic retinopathy, Glaucoma, age-related macular degeneration (AMD), as well as hypertensive retinopathy. ODIR is partitioned into subsets for testing, validation, and training purposes. It aids in optimizing and assessing algorithms, resulting in progress in automated illness diagnosis and enhancing therapies for patients in the field of ophthalmology.

### Image processing

To tackle the difficulties presented by the diverse resolutions and extensive dimensions of Diabetic Eye Disease (DED) photographs, several pre-processing measures are required. DED photos are accessible in various resolutions, such as 4288 × 2848, 4752 × 3168, 3456 × 2304, 3126 × 2136, 2896 × 1944, and 2816 × 1880. Efficiently handling the various resolutions and large dimensions of input pictures is of utmost importance, as they might result in less accurate segmentation and increased training duration for the Fully Convolutional Encoder-Decoder Network (FCEDN) model. The training and testing photos are resized to optimize them for input into the FCEDN model. The resizing process employs the bilinear interpolation technique [17] to ensure that the images are uniformly and efficiently resized to a smaller size while preserving their original aspect ratio. This stage guarantees uniformity and effectiveness throughout the training and processing of the model.

A median filter is also used to improve the quality of the pictures and make them suitable for efficient segmentation. This filter efficiently eliminates noise from the photos, which may otherwise alter the model’s interpretation of the data. Furthermore, contrast-limited adaptive histogram equalization (CLAHE) is used. CLAHE enhances the contrast of the pictures, facilitating the FCEDN model in distinguishing and segmenting different features and details in the photos. The pre-processing processes of resizing, noise reduction, and contrast enhancement are essential for improving the segmentation performance of the FCEDN model. In addition, they decrease the model’s training time, enhancing the process’s overall efficiency. By applying these processes, managing picture resolution and size variations is efficiently achieved, guaranteeing that the FCEDN model gets data properly prepared for correct segmentation and analysis.

### Data augmentation

Computer-Aided Diagnosis (CAD) technologies are designed to detect lesions linked to Diabetic Eye Disease (DED). Despite progress, these systems often face difficulties, especially regarding the high occurrence of false-positive detections in individual photos. The two primary challenges in improving the precision of lesion identification and training deep learning models are the need for human feature engineering and the limited availability of labeled data. Furthermore, fundus picture databases sometimes have size limitations and are subject to privacy problems, further complicating the training process.

To address these difficulties, this work presents a new method of enhancing fundus pictures using various strategies to improve the training dataset. The data augmentation techniques employed in this study encompass geometric transformations and patch extraction. These strategies are specifically developed to augment the variety and quantity of picture examples, hence improving the dataset used to train the CAD systems.

Geometric modifications alter pictures to simulate various viewing angles and situations, whereas patch extraction concentrates on some regions of the images, offering comprehensive perspectives of essential characteristics. The work seeks to resolve the challenges of insufficient data and enhance the model’s accuracy in identifying lesions in fundus pictures by using various data augmentation techniques. Multiple approaches have been identified and implemented with specific parameter settings to explore diverse data augmentation techniques for enhancing invariance features. The sharpening technique is applied with intensity levels of 0.5, 1, 1.5, and 2 to refine image details. Similarly, embossing is used with the same intensity levels to create a three-dimensional effect on images. Gaussian blur, known for smoothing image textures, is utilized at four scales: 0.25, 0.5, 1, and 2. Rotation is another critical technique performed at angles of 45, 90, 135, and 180 degrees to assess rotational invariance. Edge identification, crucial for highlighting outlines, is executed at four incremental intensity levels: 0.25, 0.5, 0.75, and 1.0. Skewing, which distorts the image in various directions, is conducted in left, right, forward, and backward orientations. Flipping images is another technique executed along the left side, right side, top, and bottom. Lastly, shearing, which shifts parts of an image along the X or Y axis, is performed explicitly at 10 degrees along both axes. Collectively, these methods contribute to a robust framework for enhancing data invariance in image processing.

To enrich the dataset for our research, we used several data augmentation approaches, resulting in a sevenfold increase in the number of photos. The methodology is shown in Table 3. Consequently, the collection grew to include 14,385 fundus photos. Out of them, a significant proportion of 10,070 photos, which accounts for 70% of the total, was designated for training. 4,316 photos, which account for 30% of the total, were allocated for review. The allocation of pictures between the training and evaluation stages guarantees a thorough approach to learning and evaluating the performance of the studied models.

**Table 3:** Description of the experimental augmented dataset.

Dataset /Cases	DR	DME	Glaucoma	Overall
IDRiD [33]	3612			3612
DR-HAGIS [34]	70	70	70	210
ODIR [35]	7917	1197	1449	10563
Overall Augmented	11599	1,267	1519	14385
Training sample	8120	887	1063	10,070
Testing sample	3479	380	456	4,316
Total Augmented Images	11599	1267	1519	14385

### Segmentation and feature extraction

The FCEDN is a sophisticated deep learning framework that consists of an encoder, responsible for down-sampling, and a decoder, responsible for up-sampling. Both the encoder and decoder are composed of several layers with distinct purposes. The encoder comprises convolutional (Conv), Rectified Linear Unit (ReLU), dropout, and max pooling (MP) layers. In contrast, the decoder consists of transposed convolutional (TC), up-sampling (UP), ReLU, and dropout (DO) layers.

Creating a functional FCEDN framework customized for specific applications might present difficulties. A combination of testing and ideas derived from previous research is often necessary [71–74]. The pertinent research papers in our investigation shape the FCEDN’s original structure. The encoder is configured with four Convolutional layers, one dropout layer, four Rectified Linear Unit (ReLU) levels, and two pooling layers. The decoder comprises four TC layers, two UP levels, four ReLU layers, and one DO layer. The Convolutional (Conv), Transposed Convolution (TC), pooling, and Upsampling (UP) layers use kernel sizes of either 3x3 or 5x5. The number of kernels in each layer varies, ranging from 20 to 200. The quantity of kernels increases as we go from the first stages to the subsequent ones. The model’s regularisation technique uses a dropout rate between 0.2 and 0.4.

The quantity of Convolutional, Temporal Convolutional, pooling, and Upsampling layers determines the general architecture of the FCEDN. Ensuring a proper balance of these layers is of utmost importance; an excessive number of convolutional layers may lead to overfitting, while an insufficient number may result in underfitting. Moreover, the amount of pooling layers influences feature representation. Having more pooling layers might remove some features, whereas having fewer layers may result in repeating features. The research establishes the minimum and maximum values for Convolutional (Conv), Temporal Convolutional (TC), pooling, and Upsampling (UP) layers as 2 and 10, respectively. Multiple trials were carried out to determine the ideal equilibrium between efficacy and computational efficiency.

Optimizing the FCEDN’s hyperparameters utilizing G-GWO may be divided into four steps: encoding, population initialization, fitness evaluation, and population updating. The encoding phase entails encoding key hyperparameters of FCEDN, such as the Conv layer. The size of the kernel in the transpose convolution operation is called C-KS. The size of the kernel (TC-KS), the number of convolutional kernels (C-NK), and the transpose convolution The number of kernels (TC-NK), maximum pooling The size of the kernel, referred to as MP-KS, and the process of unpooling. The kernel size (UP-KS) and dropout rate (DL-Dr) are transformed into a k-dimensional vector inside the dropout layer. The elements in this vector are selected randomly from a pre-established range. The i-th parameter vector is officially represented by Eq (16).


$$P_{i}=\{R_{i1},R_{i2},R_{i3}\ldots .R_{ik}\}$$ (16)

The Fully Convolutional Encoder-Decoder Network (FCEDN) architecture consists of a total of four convolution (Conv) layers, two dropout (DL) layers, two max-pooling (MP) layers, four transposed convolution (T-Conv) layers, and two up-sampling (UP) layers. Based on this arrangement, the vector size (k) required to describe the hyperparameters of these layers is calculated to be 22. This vector size represents the different hyperparameters linked to each layer in the network. The hyperparameters that correspond to the elements of this vector are as follows: C1-Nk&Ks, C2-Nk&Ks, MP1-Ps, DL1-Dr, C3-Nk&Ks, C4-Nk&Ks, MP2-Ps, DL2-Dr, UP1-ps, TC1-Nk&Ks, TC2-Nk&Ks, UP1-ps, TC3-Ks&Nk, TC4-Ks&Nk.

To commence the optimization process, a set of n encoding vectors, abbreviated as Xn, is constructed to represent the initial population of grey wolves. Each vector Xi in this population represents the location of the ith grey wolf in the search space and is a k-dimensional vector that encodes the FCEDN hyperparameters.

A streamlined model is trained using fewer, randomly selected samples to accelerate the fitness assessment process and save computing time. This method guarantees prompt detection of even the slightest changes in the fitness value. The coefficient vectors n ⃑, N ⃑, and Q ⃑ in the G-GWO are formed using Eqs (2), (3) and (10) correspondingly.

After this configuration, the fitness of every agent in the population is assessed. The method updates the overall population while preserving the positions of the top three agents (α, β, and δ) for a certain number of iterations. The procedure is outlined in the given pseudocode. Ultimately, the agent with the highest fitness value signifies the most practical combination of hyperparameters for the FCEDN.

This work aims to optimize the hyperparameters of the FCEDN model for picture segmentation. The objective function for this optimization, driven by the G-GWO, is formulated to maximize the Jaccard coefficient. The Jaccard coefficient, which quantifies the similarity and diversity, is a crucial indicator for evaluating the efficacy of the selected hyperparameters in the segmentation job.


$$f(X_{i})=1/tim\left(\sum_{m=1}^{tim}\left(\frac{\varepsilon +\sum_{j=1,l=1}^{j=r,l=c}y_{m}(j,l){\hat{y}}_{m}(j,l)}{\varepsilon +\sum_{j=1,l=1}^{j=r,l=c}y_{m}(j,l)+\varepsilon +\sum_{j=1,l=1}^{j=r,l=c}{\hat{y}}_{m}(j,l)-\varepsilon +\sum_{j=1,l=1}^{j=r,l=c}y_{m}(j,l){\hat{y}}_{m}(j,l)}\right)\right)$$ (17)

Our research aims to assess the precision of picture segmentation at the pixel level using the Fully Convolutional Encoder-Decoder Network (FCEDN). For every pixel in the mth picture with dimensions (r*c), ’y_m (j,l)’ represents the actual pixel value at location (j,l), whereas ’y^_m (j,l)’ means the projected label for the same pixel, based on the position vector Xi derived by FCEDN. A random smoothness value between 0 and 1 is selected to simplify this procedure, and ’tim’ denotes the fraction of pictures utilized for training. In evaluating the enhanced architectural configurations of the Fully Enhanced Convolutional Deep Network (FECDN), Table 4 presents the revised parameter settings, showcasing adjustments to optimize network performance.

**Table 4:** Configuration of FCEDN.

Configuration Aspect	Setting
Convolutional Layers (Conv)	5 layers
Activation Layers (ReLU)	5+3 configuration
Convolutional Filters (Conv_K)	25, 55, 75, 105 counts
Size of Convolutional Kernels (Conv_K Size)	3x3 for each layer
Transitional Convolution (TC) Layers	5 layers
Transitional Convolutional Filters (TC_K)	75, 55, 25, 5 counts
Size of TC Kernels (TC Size)	3x3 for all, except last at 2x2
Pooling Layers	3 layers
Pooling Kernel Sizes (Pooling_K)	2x2 for each
Upsampling (UP) Layers	3 layers
Upsampling Kernel Sizes (UP_K Size)	2x2 for each
Dropout Rates (DO_Rate)	0.25

An essential difficulty in segmentation jobs, particularly those with a small number of classes, is the problem of class imbalance. In such instances, a deep neural network may get a seemingly high level of accuracy, such as 80%, by accurately detecting the majority of background pixels. Nevertheless, this does not always indicate precise division since the crucial focus regions often constitute a smaller fraction of the picture, roughly 20%. Hence, relying just on accuracy as the criteria for assessing segmentation performance might be deceptive. A more suitable metric is the percentage of overlap between the actual masks and the anticipated masks. The measure is derived by computing the intersection of pixels between the forecast and ground truth masks and then dividing this by the total number of pixels in both datasets. This methodology offers a more precise evaluation of the model’s efficacy in delineating the specific regions of interest within the picture. Using this assessment technique, we may enhance our comprehension of the model’s effectiveness in segmentation tasks, guaranteeing its precise identification and differentiation of the regions of interest rather than only focusing on the prominent background pixels.

Expanding on the effective segmentation outcomes, this research subsequently concentrated on meticulously examining fundus pictures (FIs) using a Convolutional Neural Network (CNN). The segmentation results established a firm basis, allowing us to explore the intricate details of these photos. This research systematically identifies the essential characteristics that may distinguish between the different phases of Diabetic Retinopathy (DR), Glaucoma, and DME. To streamline this process, we selected a Convolutional Neural Network (CNN) model that was simple and efficient, primarily focusing on accentuating distinct attributes. Using the method shown in Fig 1, smoothly shift from the segmentation stage to a more focused feature extraction stage. Every layer of our Convolutional Neural Network (CNN) has a crucial function in breaking down the segmented pictures and focusing on the essential elements necessary for accurately diagnosing the stages of Diabetic Retinopathy (DR), Glaucoma, and Diabetic Macular Edema (DME). We improved this approach by using batch normalization, ensuring consistent input distribution across layers, simplifying training, and enhancing the model’s generalization capacity.

Additionally, max-pooling concentrated and condensed the segmented pictures to their most prominent characteristics. Nevertheless, because of the intricate nature of more complicated networks and the abundance of parameters, there was a potential danger of excessively tailoring the training data. Dropout layers addressed this issue by randomly deactivating neurons during training sessions. This approach helps prevent overreliance on specific characteristics, fostering a more universal and efficient learning experience. After optimizing the training process using the Adam optimizer, specifically selected for its effectiveness in managing large datasets such as our FIs collection, we successfully reached the final step of our model. A densely connected layer extracts distinct features from each split feature image in this case. Subsequently, Singular Value Decomposition (SVD) was used to reduce the dimensionality, ensuring that only the most essential data patterns were preserved. This stage was vital in transferring from dividing our information into segments to categorizing it, intending to simplify the dataset to its fundamental elements while protecting the essential attributes necessary for classifying DR, Glaucoma, and DME.

### KELM hyperparameter optimization using G-GWO for classification

This paper presents a novel computational framework called GGWO-KELM, particularly for medical diagnostics. The GGWO-KELM framework consists of two main stages, both essential for achieving precise and efficient diagnosis using medical data.

The first phase utilizes the G-GWO) method. The primary goal of this step is to analyze the medical data and remove any unnecessary or unrelated information. This approach entails dynamically looking for the data’s most pragmatic combination of characteristics. The GGWO technique commences with the genetic technique (GA) to produce the starting placements of the population inside the feature space. Afterward, the GWO approach repeatedly updates these locations inside a discrete search space. This technique enables the identification of traits that are essential for precise diagnosis. After selecting the most relevant features, the next step is implementing the Kernel Extreme Learning Machine (KELM) classifier. The classifier is used on the refined feature subset acquired from the first step. The KELM classifier is renowned for its efficacy and efficiency, making it a perfect selection for processing the optimized feature set and generating diagnostic outputs.

Fig 4 presents a detailed flowchart of the GGWO-KELM framework. This flowchart visually depicts every step involved in the two-phase process. The main objective of the GGWO in this architecture is to dynamically explore the feature space, determining the combination of features that optimizes classification accuracy while decreasing the number of needed features.

The efficacy of the chosen characteristics is evaluated by using a fitness function inside the GGWO. This fitness function assesses the features by considering their impact on classification accuracy and quantity, guaranteeing that the resulting feature set is concise and effective in generating accurate medical diagnoses. Using a dual-phase methodology inside the GGWO-KELM framework signifies a notable breakthrough in using computational techniques for medical diagnostic applications.


$$Fitness=\alpha P+\beta \frac{N-L}{N}$$ (18)

The Fitness formula integrates many essential components:

P: Denotes the precision of the categorization model. It is a crucial measure that indicates the model’s accuracy inappropriately categorizing the data.

The symbol "L" represents the length of the chosen subset of features. The size refers to the number of features selected during the selection procedure.

N: The overall count of features included in the dataset. This Figure serves as a point of reference for comprehending the relative size of the chosen features.

*α* and *β* are parameters that determine the trade-off between the classification accuracy and the quality of feature selection. The variable *α*, bounded between 0 and 1, directly impacts the weighting assigned to classification accuracy. On the other hand, *β*, which is calculated as 1−α, modifies the importance placed on the quality of feature selection.

The process of selecting features is shown using a flag vector. The vector comprises binary values (0s and 1s), with each value indicating the selection status of a feature in the dataset. In this vector, a ’1’ denotes the inclusion of the associated trait, while a ’0’ indicates its exclusion. The size of the feature subset is determined by the total number of chosen features, represented by ’1’s in the vector.

In a problem with ’*n*’ dimensions, the flag vector consists of ’*n*’ bits. Each bit represents a specific characteristic in the dataset, with the *i*th bit (*i* = 1, 2,…, *n*) corresponding to the *i*th feature. The binary format facilitates the monitoring of the characteristics used in the model.

Additionally, the report has a pseudocode (pseudocode 1) that comprehensively describes the G-GWO method. The provided pseudocode systematically explains the IGWO algorithm’s functioning, elucidating the feature selection and optimization process used in the classification model. By including this pseudocode, a precise and straightforward framework is provided to comprehend the computational mechanics involved in feature selection and model optimization.

### Pseudocode 1 contains the G-GWO algorithm’s pseudocode

**Initialization:**

1. Define Key Variables:

   - Population size (`popsize`), maximum iterations (`maxiter`), feature dimensionality (`dim`), grey wolf positions (`GWs[pos]`), feature selection marker (`flag[]`).

2. Generate Initial Grey Wolf Positions:

   - Use Genetic Algorithm (GA) for initial `GWs[pos]` setup.

   - Initialize variables `n,` vector `N,` and vector `Q.`

**First Population Assessment:**

1. Feature Selection:

   - For each grey wolf `i` and each feature `j`:

     - If `pos[i, j]` > random number, then `flag[j] = 1` (feature selected).

     - Else, `flag[j] = 0` (feature not selected).

2. Fitness Evaluation:

   - Calculate fitness of each GW using `αZ + β(H-G)/H`.

   - Identify the top three GWs: alpha (α), beta (β), and delta (δ).

**Iterative Optimization:**

1. Loop Until Max Iterations:

   - For each iteration `k < maxiter`:

     - For each grey wolf `i`:

       - Update `GWs[pos]` using Eq 10.

       - For each feature `j`:

         - If `pos[i, j]` > random integer, `flag[j] = 1`.

         - Else, `flag[j] = 0`.

       - Update variables `n,` `N,` and `Q.`

       - Recalculate fitness using Eq 11.

       - Update rankings of α, β, and δ.

       - Increment iteration counter `k.`

**Conclude Optimization:**

1. Final Feature Set Determination:

   - Upon reaching `maxiter,` return the best feature set from the alpha grey wolf.

The GGWO algorithm employs a systematic technique to find the most attractive feature subset for the given goal, as elaborated in the works of Albadr et al. [75,76]. The algorithm progressively improves the location of each GWO in the feature space by assessing and modifying their fitness according to a predetermined fitness function. The result is the identification of an ideal collection of characteristics chosen by the dominant wolf that most effectively meets the criteria of the classification model.

To refine the classification accuracy of Diabetic Eye Disease detection, we systematically optimized the Fully convolutional Encoder-Decoder Network (FCEDN). Parameters This optimization was achieved by integrating G-GWO and further enhancements using the Kernel Extreme Learning Machine (KELM). Table 5 compares the parameter configurations across the original FCEDN model, its enhancement through G-GWO, and subsequent optimization with G-GWO-KELM. The comparative overview illustrates our methodological approach to achieving superior classification performance. The specific parameter settings utilized in our experiments are detailed below the table, providing insights into the optimization process and its impact on the model’s effectiveness in classifying Diabetic Eye Disease.

**Table 5:** Comparative overview of parameter configurations for FCEDN, enhanced with G-GWO and G-GWO-KELM optimizations.

Setting	Description
Population Count	8
Iteration Quantity	Extended to 200 iterations for an exhaustive search
Feature Count (n)	Represents the comprehensive number of features evaluated
Domain Range	Broadened to the interval [–1, 2] for broader search space
GA Crossover Rate	0.8
GA Mutation Rate	0.01
Fitness Function Alpha (α)	Adjusted importance to 0.99 for balancing precision and diversity
Fitness Function Beta (β)	Adjusted to a complementary weighting of 0.01
KELM Regularization (C)	Increased to 32 for improved regularization
KELM Kernel Parameter (γ)	Adjusted to a value of 0.5 for optimal kernel performance

### Performance metrics

When it comes to machine learning, it is crucial to evaluate the efficacy of a model in real-world scenarios, going beyond its performance during the initial training phase. For comprehensive details on the equations used in our evaluation metrics, which bolster our analysis and understanding of model performance across diverse conditions, readers are referred to [77–80]. This entails using diverse assessment criteria, starting with essential indicators for categorization. Sensitivity, or the True Positive Rate, quantifies the model’s capacity to detect positive instances accurately. Conversely, Specificity, often known as the True Negative Rate, measures the model’s ability to identify virtual negative occurrences correctly. Accuracy provides a comprehensive measure of the model’s total capacity to accurately align its predictions with the actual results, including both classes. The F1-Score, which combines accuracy and recall, is especially important in cases when the distribution of classes is not balanced. Matthew’s Correlation Coefficient (MCC) offers a fair assessment that considers the differences in class sizes. Precision is concerned with the accuracy of optimistic forecasts, whereas the Error Rate quantifies the fraction of inaccurate forecasts. The purpose of these measures is to provide a comprehensive, unambiguous, and accurate assessment of the model’s performance. The mathematical equations for these measurements are outlined in Table 6 to give a complete comprehension.

**Table 6:** Performance evaluation matrices.

Matric Name	Mathematical Representation
Accuracy (ACC)	$\frac{TP+TN}{TP+TN+FP+FN}\times 100\%$
Sensitivity (SEN)	$\frac{TP}{TP+FN}\times 100\%$
Specificity (SPC)	$\frac{TN}{TN+FP}\times 100\%$
Precision (PRE)	$\frac{TP}{TP+FP}\times 100\%$
MCC	$\frac{TP\times TN-FP\times FN}{\sqrt{\left(TP+FP)(TP+FN)(TN+FP)(TN+FN\right)}}\times 100\%$
Error Rate (ER)	$1-0.5\times \frac{Sensitivity\;value+Specifity\;values}{100}\times 100\%$
F1-Score (F1-S)	$\frac{2\times Precision\times Recall}{Precision+Recall}\times 100\%$

## Results and discussion

### Results

**Fig 6:**
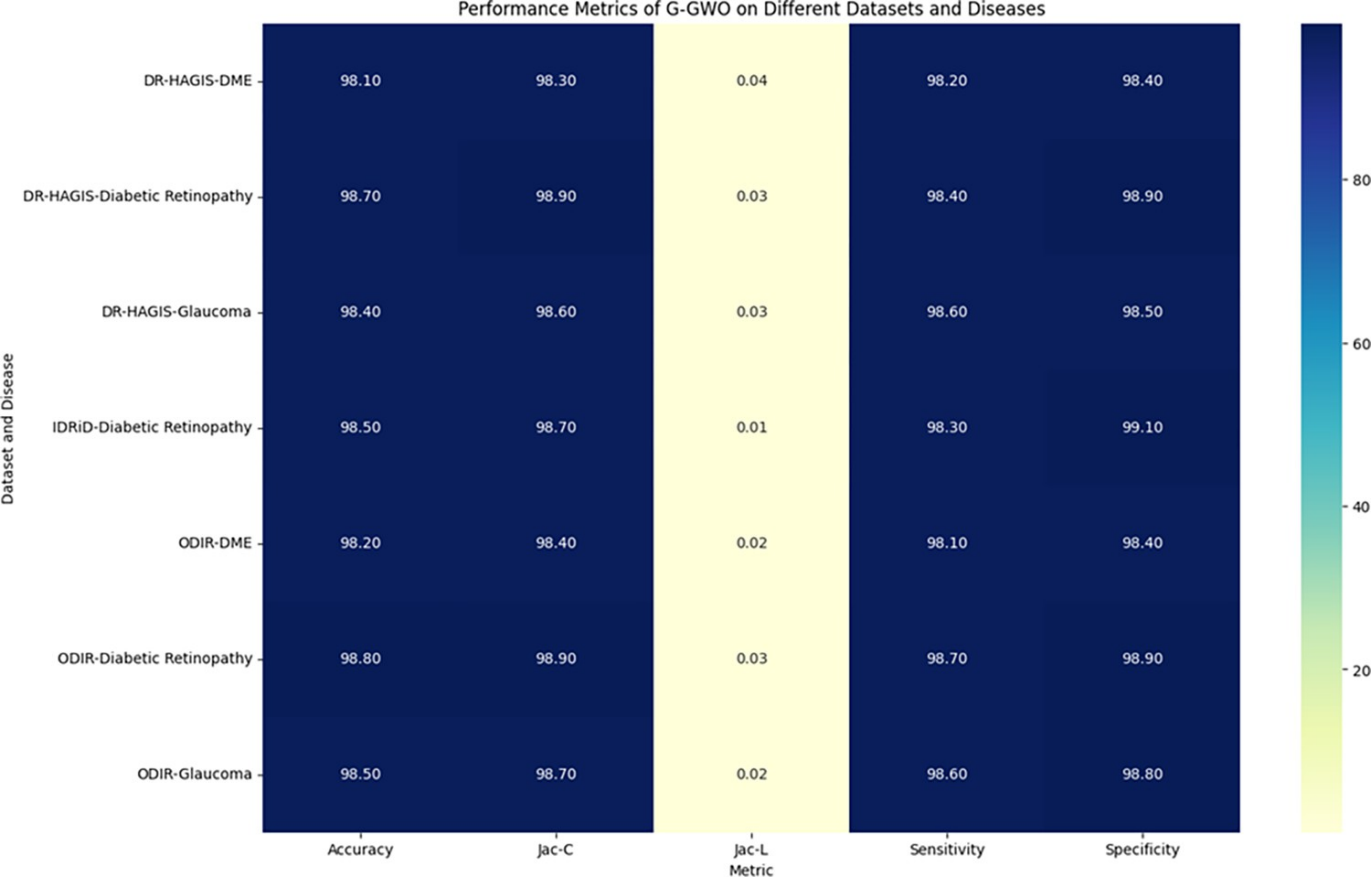
Segmentation performance matrices comparison.

This research assesses the effectiveness of the FCEDN model in attaining accurate segmentation. The FCEDN architecture consists of four convolutional layers with Rectified Linear Unit (ReLU) activation, two Max Pooling (MP) layers, two Dropout (DO) levels, four Transposed Convolution (TC) layers, and two Upsampling (UP) layers. The convolutional layers consist of 20, 50, 70, and 100 filters, each with a consistent size of 4. Conversely, the TC layers include filters with dimensions of 70, 50, 20, and 2, respectively. The diameters of these filters are 4, 4, 4, and 2 units, correspondingly. The model uses a particle size of two and a dissolved oxygen rate 0.2 in both the MP and UP layers. The last layer of the Fully Convolutional Encoder-Decoder Network (FCEDN) comprises a Transition Convolution (TC) layer with two filters, each two in size. These filters are specifically intended to capture the distinctive aspects of the picture precisely. The result is generated using the softmax activation method. The FCEDN model employs the Adam optimizer, using a constant learning rate of 0.001 and a batch size 20 throughout the training phase. The FCEDN architecture is selected based on previous research and comprises various arrangements of convolutional (conv), rectified linear unit (ReLU), max pooling (MP), transposed convolution (TC), and upsampling (UP) layers. The number and dimensions of these layers and the number of filters in the convolutional and transposed convolution layers are modified in various simulations. However, the other network characteristics remain unchanged despite these modifications. The FCEDN architecture consists of four Convolutional layers, eight Rectified Linear Unit (ReLU) levels, two Dropout (DO) layers, two Max Pooling (MP) layers, four Transposed Convolution (TC) layers, and two Upsampling (UP) layers. The models were subjected to 500 iterations of testing using the IDRiD, DR-HAGIS, and ODIR datasets. The Jaccard coefficient and Jaccard loss were calculated for each training iteration, and the results were analyzed, focusing on the model’s ability to segment the datasets accurately, as seen in Fig 6.

**Fig 7:**
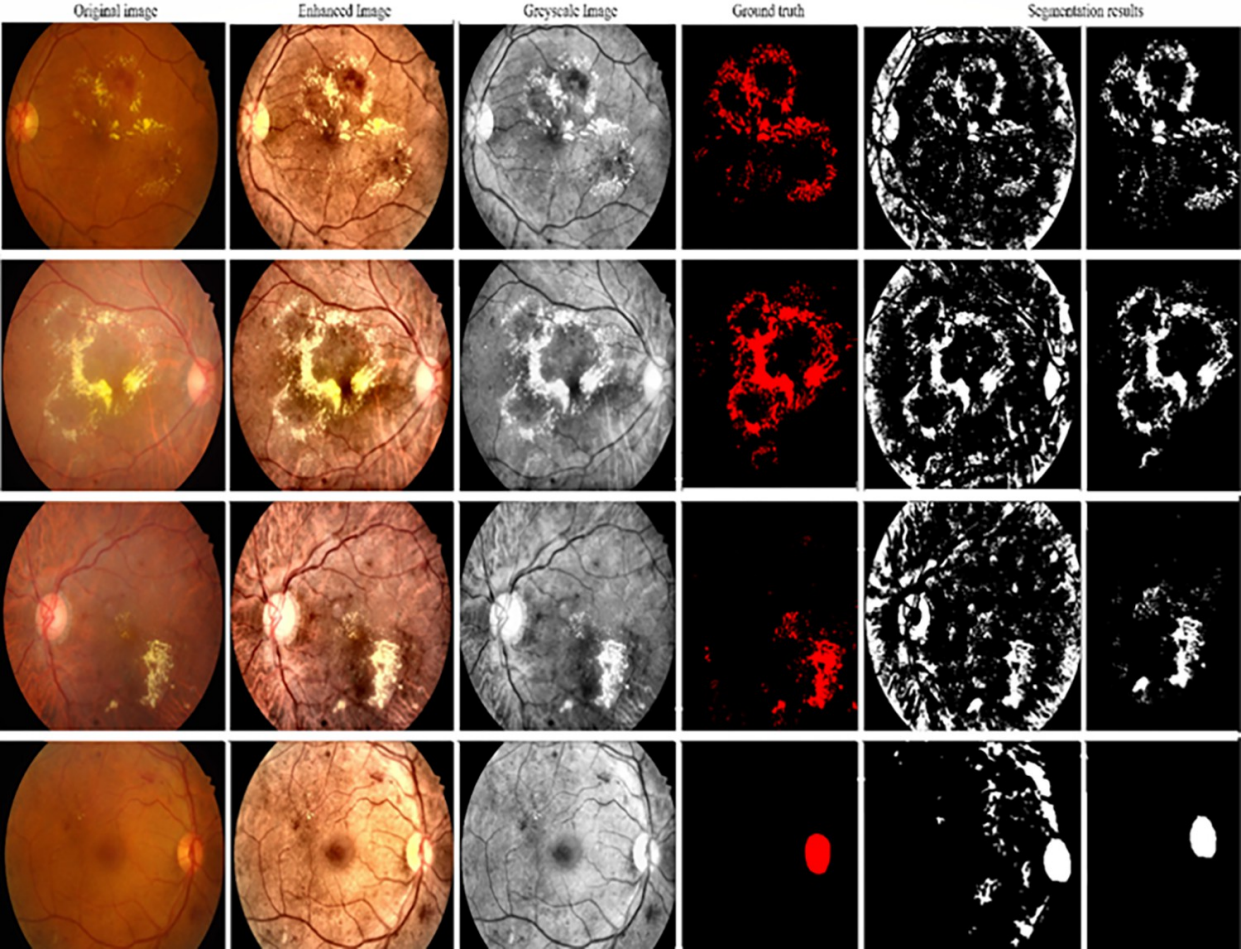
Input, pre-processing, ground truth, and the predicted segmentation results were obtained by the proposed model for some sample images.

Fig 7 depicts the step-by-step procedure, beginning with the original input, then pre-processing, and concluding with presenting the ground truth and the predicted masks produced by the FCEDN model for several sample photos. The illustrations show successful segmentation of the specific region by the model.

This study explores sophisticated classification methods using the GGWO-KELM model, renowned for its exceptional computer vision skills. The study’s fundamental purpose is to showcase the efficacy of the new GGWO-KELM algorithm. This method enhances performance by combining the GWO and Genetic algorithm. A wide range of assessment indicators is used to evaluate the effectiveness and dependability of the GGWO-KELM model. The evaluation metrics used in this study include the training set mean square error, testing accuracy, ROC curve analysis, confusion matrix, and critical statistical indicators such as sensitivity, Specificity, precision, Matthews correlation coefficient (MCC), error rate, and the F1-score. This comprehensive evaluation methodology thoroughly examines the model’s precision, coherence, and robustness across many assessment criteria.

Table 7 thoroughly assesses four distinct algorithms (KELM, GA-KELM, GWO-KELM, GGWO-KELM) for diagnosing Diabetic Retinopathy. The performance of each algorithm is evaluated based on various critical indicators. The GGWO-KELM algorithm routinely demonstrates superior performance. It has the most remarkable accuracy, sensitivity, Specificity, precision, Matthews Correlation Coefficient (MCC), and F1-Score among all the algorithms. GGWO-KELM has a remarkable accuracy rate of 98.6%, highlighting its extraordinary capacity for accurate classification. The sensitivity of 97.2% demonstrates its ability to reliably identify positive situations, while its Specificity of 99.0% indicates its skill in correctly detecting negative cases. The accuracy of GGWO-KELM is 98.5%, highlighting its capacity to provide accurate and optimistic forecasts. The MCC value of 0.984 signifies the robustness of the classification, while the error rate of just 0.014 showcases its high accuracy. The F1-Score of 0.987 demonstrates its efficacy in balancing accuracy and recall.GGWO-KELM regularly surpasses the other algorithms in several crucial measures, giving it an up-and-coming option for detecting Diabetic Retinopathy in this dataset. Its outstanding accuracy, precision, and robust MCC and F1-Score make it the top performer for this assignment.

**Table 7:** Different classifiers Performance evaluation using IDRiD dataset.

Dataset	Disease	Metric	KELM	GA-KELM	GWO-KELM	GGWO-KELM
IDRiD	Diabetic Retinopathy	Accuracy	88.5	82.7	84.1	98.6
		Sensitivity	85.8	87.2	86.3	97.2
		Specificity	89.1	76.9	81.0	99.0
		Precision	87.9	82.1	83.4	98.5
		MCC	0.742	0.665	0.695	0.984
		Error Rate	0.112	0.173	0.158	0.014
		F1-Score	0.881	0.854	0.867	0.987

**Fig 8:**
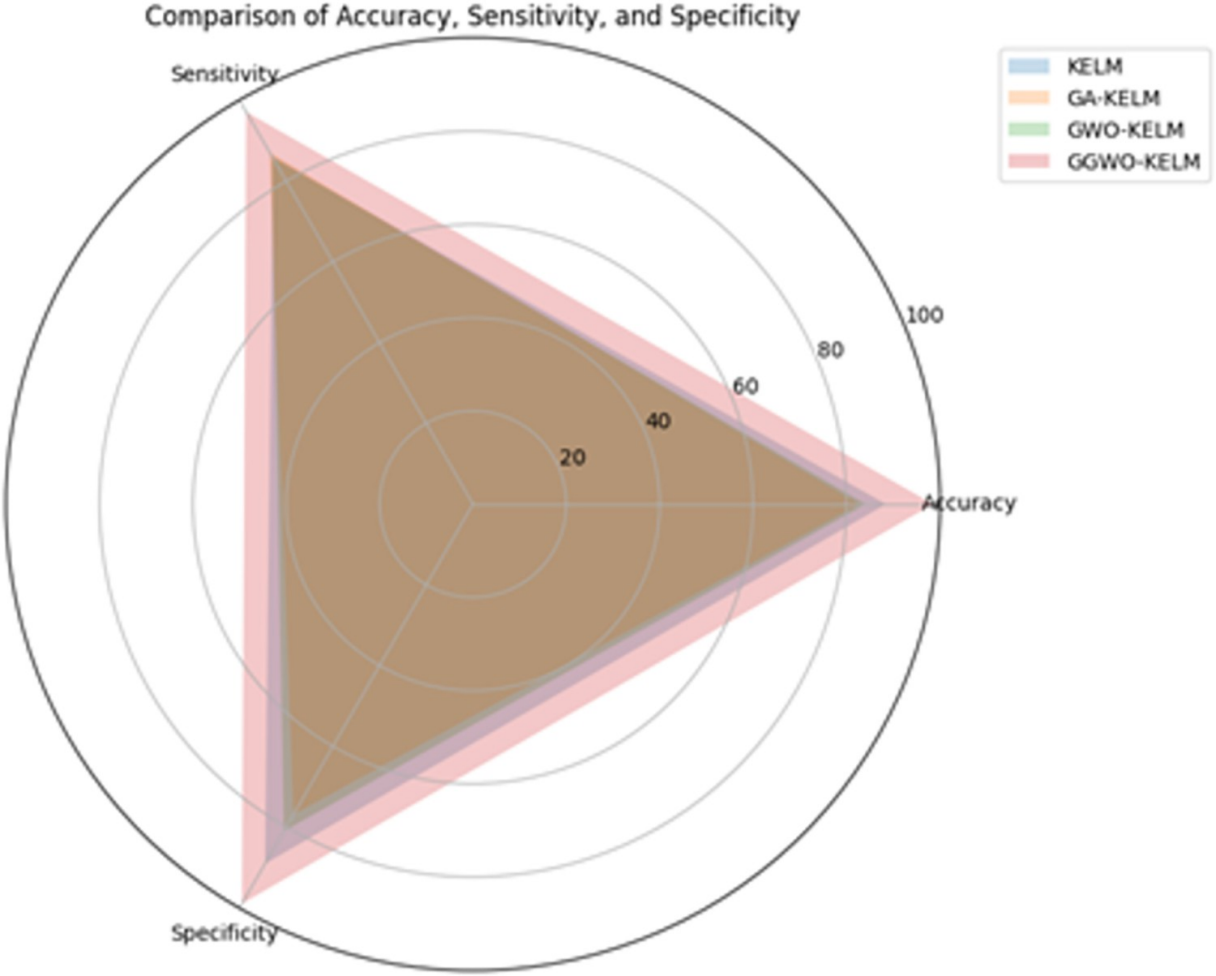
ACC, SEN, and SPC evaluation using IDRiD dataset.

Fig 8 presents an evaluation of Accuracy, Sensitivity, and Specificity to examine the performance of four different algorithms (KELM, GA-KELM, GWO-KELM, and GGWO-KELM) in detecting Diabetic Retinopathy. GGWO-KELM stands out as the undisputed frontrunner in accuracy, with an impressive score of about 98.6%. This makes it the most precise algorithm out of the four options. The GGWO-KELM method has a high sensitivity of around 97.2%, indicating its ability to detect positive cases accurately. Furthermore, GGWO-KELM demonstrates exceptional Specificity by successfully identifying negative instances with an approximate rate of 99.0%. On the other hand, GA-KELM shows the least favorable results in these three measurements, with an accuracy of around 82.7%, a sensitivity of 87.2%, and a specificity of 76.9%. GGWO-KELM exhibits greater diagnostic accuracy for Diabetic Retinopathy, but GA-KELM falls behind in overall correctness.

**Fig 9:**
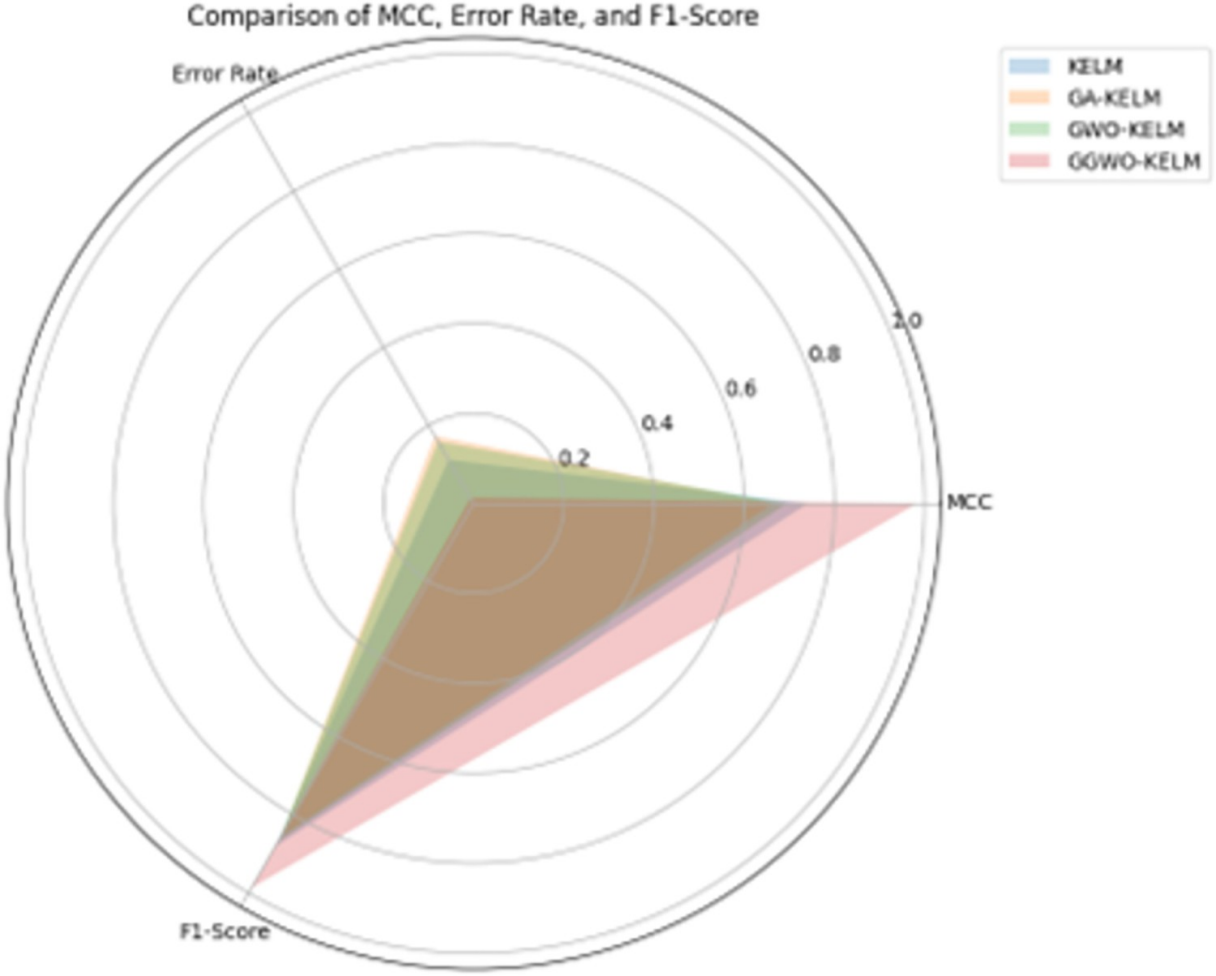
MCC, ER, and F1-Score evaluation using IDRiD dataset.

Fig 9 examines explicitly more sophisticated assessment criteria, such as the Matthews Correlation Coefficient (MCC), Error Rate, and F1-Score, to provide a more comprehensive review of the performance of the same four algorithms. GGWO-KELM maintains its exceptional performance, with a remarkable MCC score of about 0.984, indicating strong performance and accurate predictions. GGWO-KELM has the most optimal performance in terms of Error Rate, with a meager value of about 0.014, signifying the least misclassifications. Furthermore, GGWO-KELM maintains its dominant position in the F1-Score, with a fantastic score of about 0.987. This demonstrates a seamless equilibrium between accuracy and responsiveness.

On the other hand, GA-KELM falls behind with the lowest MCC score, the most excellent Error Rate, and a trade-off in F1-Score. To summarize, these spider plots thoroughly assess algorithm effectiveness in detecting Diabetic Retinopathy, with GGWO-KELM constantly surpassing its competitors in performance. Simultaneously, GA-KELM demonstrates the potential for enhancement in its diagnostic skills.

**Fig 10:**
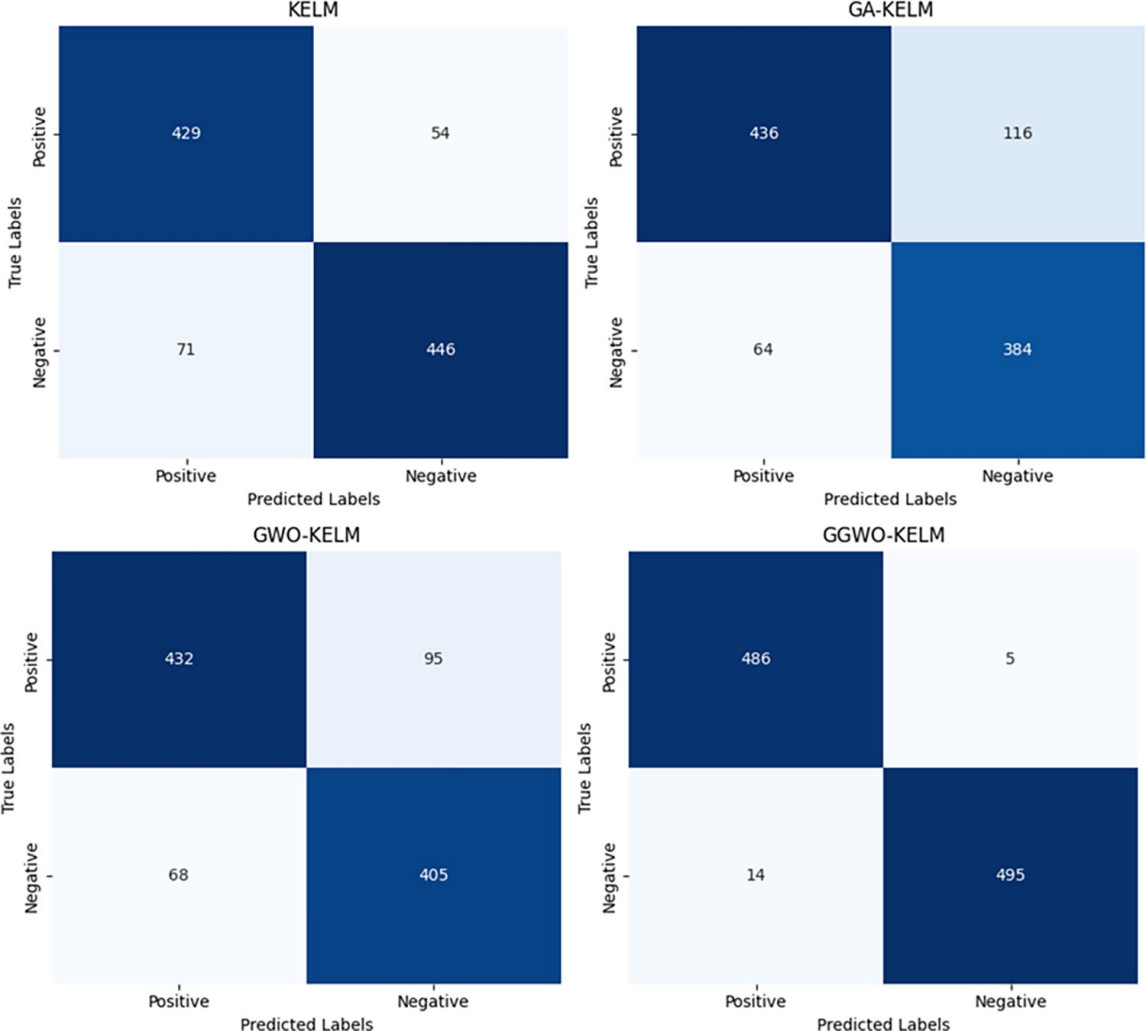
Confusion matrix comparison of four algorithms on IDRiD dataset for diabetic retinopathy, showcasing GGWO-KELM’s exceptional performance.

Incorporating the results presented in the Fig 10, the visual comparison reinforces the textual analysis, vividly illustrating the unmatched precision of GGWO-KELM in accurately classifying Diabetic Retinopathy. This graphical representation complements the discussion and provides a clear, at-a-glance understanding of each algorithm’s performance, further substantiating GGWO-KELM’s superiority in achieving the most favorable balance between identifying actual cases and reducing errors.

Table 8 presents a thorough analysis of three disease categories (Diabetic Retinopathy, Diabetic Macular Edema—DME, and Glaucoma) using four different algorithms (KELM, GA-KELM, GWO-KELM, GGWO-KELM). The analysis focuses on several essential performance metrics.

**Table 8:** Different classifiers performance evaluation using DR-HSGIS dataset.

Dataset	Disease	Metric	KELM	GA-KELM	GWO-KELM	GGWO-KELM
DR-HAGIS	Diabetic Retinopathy	Accuracy	88.9	83.0	83.3	97.6
		Sensitivity	86.4	87.8	86.1	96.3
		Specificity	89.7	78.1	80.2	98.8
		Precision	88.6	81.4	82.0	97.7
		MCC	0.757	0.680	0.696	0.982
		Error Rate	0.102	0.167	0.162	0.015
		F1-Score	0.889	0.862	0.873	0.986
DR-HAGIS	DME	Accuracy	87.9	82.0	82.6	98.1
		Sensitivity	84.9	86.7	85.5	98.0
		Specificity	88.6	77.8	80.1	98.3
		Precision	87.1	80.9	81.4	98.4
		MCC	0.729	0.647	0.668	0.979
		Error Rate	0.123	0.184	0.174	0.018
		F1-Score	0.872	0.854	0.865	0.982
DR-HAGIS	Glaucoma	Accuracy	88.7	91.3	82.1	98.4
		Sensitivity	87.3	90.0	91.2	98.6
		Specificity	89.2	92.1	92.6	98.3
		Precision	87.7	91.0	91.8	98.5
		MCC	0.759	0.837	0.849	0.978
		Error Rate	0.112	0.095	0.087	0.016
		F1-Score	0.882	0.903	0.914	0.986

Among the algorithms evaluated for Diabetic Retinopathy (DR-HAGIS), GGWO-KELM consistently demonstrates superior performance. With an impressive accuracy rate of 97.6%, it has exceptional categorization skills. GGWO-KELM shows a high level of sensitivity (96.3%), Specificity (98.8%), accuracy (97.7%), Matthews correlation coefficient (MCC) of 0.982, and F1-Score of 0.986. These results emphasize its exceptional performance in all aspects. GGWO-KELM again demonstrates its outstanding skill in reliably differentiating instances of Diabetic Macular Edema (DME), with an impressive accuracy rate of 98.1%. The method has strong performance in terms of sensitivity (98.0%), Specificity (98.3%), precision (98.4%), Matthews correlation coefficient (MCC) (0.979), and F1-score (0.982), establishing it as the top algorithm in this illness category. GGWO-KELM performs exceptionally well in Glaucoma, with an accuracy rate of 98.4%. This high level of accuracy underscores its efficacy in the categorization process. The GGWO-KELM has outstanding sensitivity (98.6%), Specificity (98.3%), accuracy (98.5%), Matthews correlation coefficient (0.978), and F1-Score (0.986), highlighting its excellent diagnostic capabilities for Glaucoma.

GGWO-KELM regularly performs better than the other algorithms in all three illness categories, highlighting its adaptability and usefulness in diverse medical diagnoses. The accuracy, sensitivity, Specificity, precision, Matthews correlation coefficient (MCC), and F1-Score continually make it the top option for precise illness categorization.

**Fig 11:**
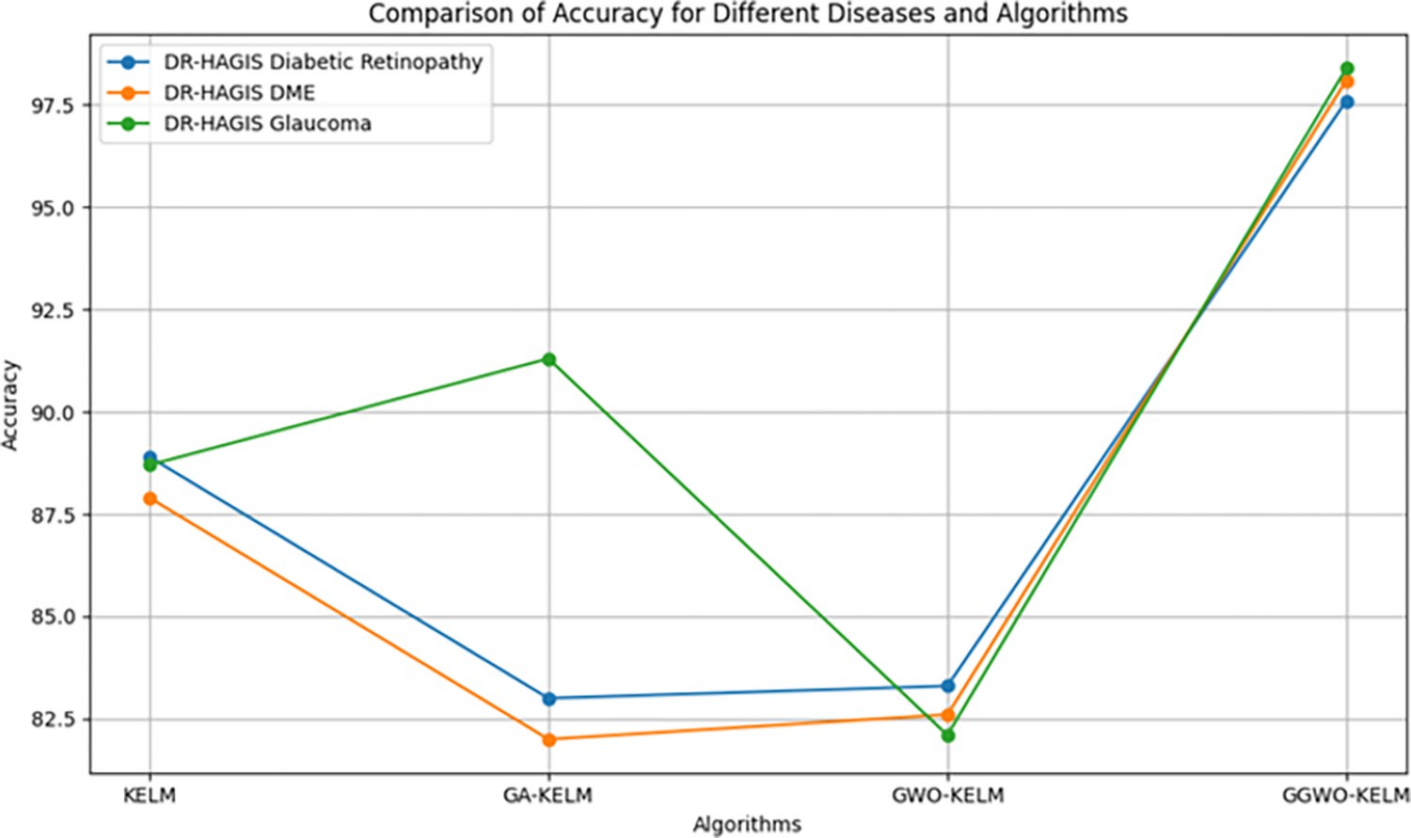
Accuracy evaluation using DR-HAGIS dataset.

**Fig 12:**
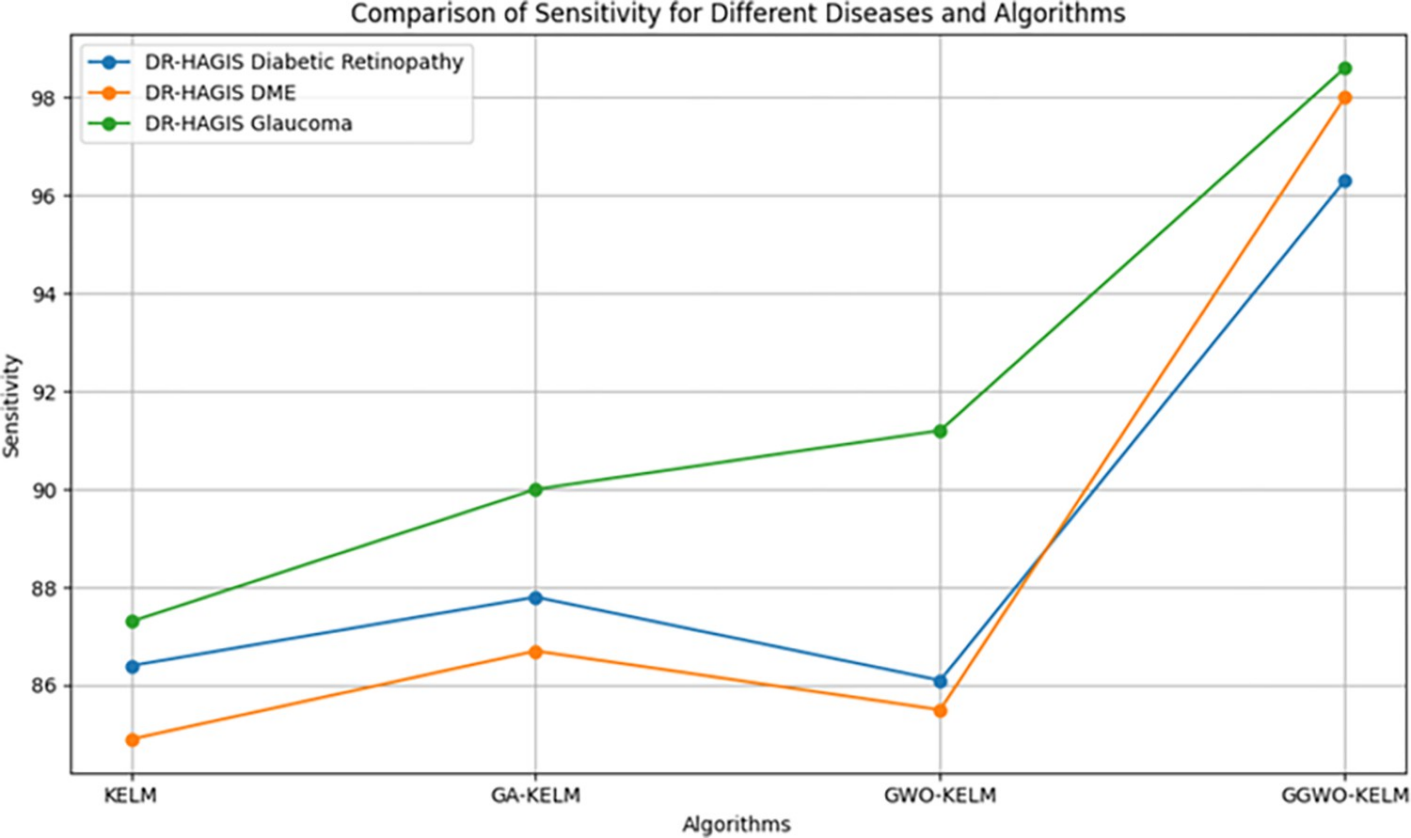
Sensitivity evaluation using DR-HAGIS dataset.

**Fig 13:**
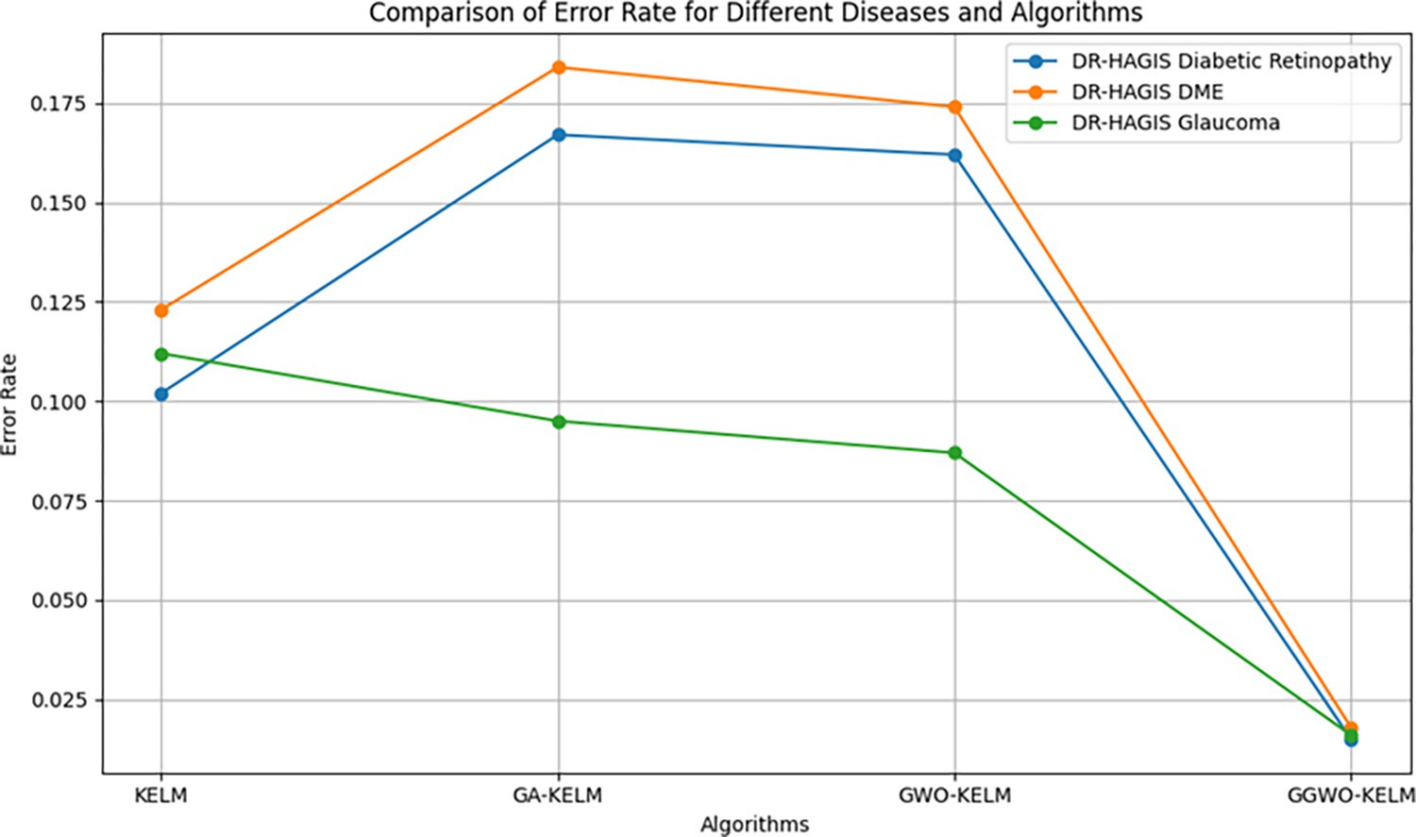
Error Rate evaluation using DR-HAGIS dataset.

**Fig 14:**
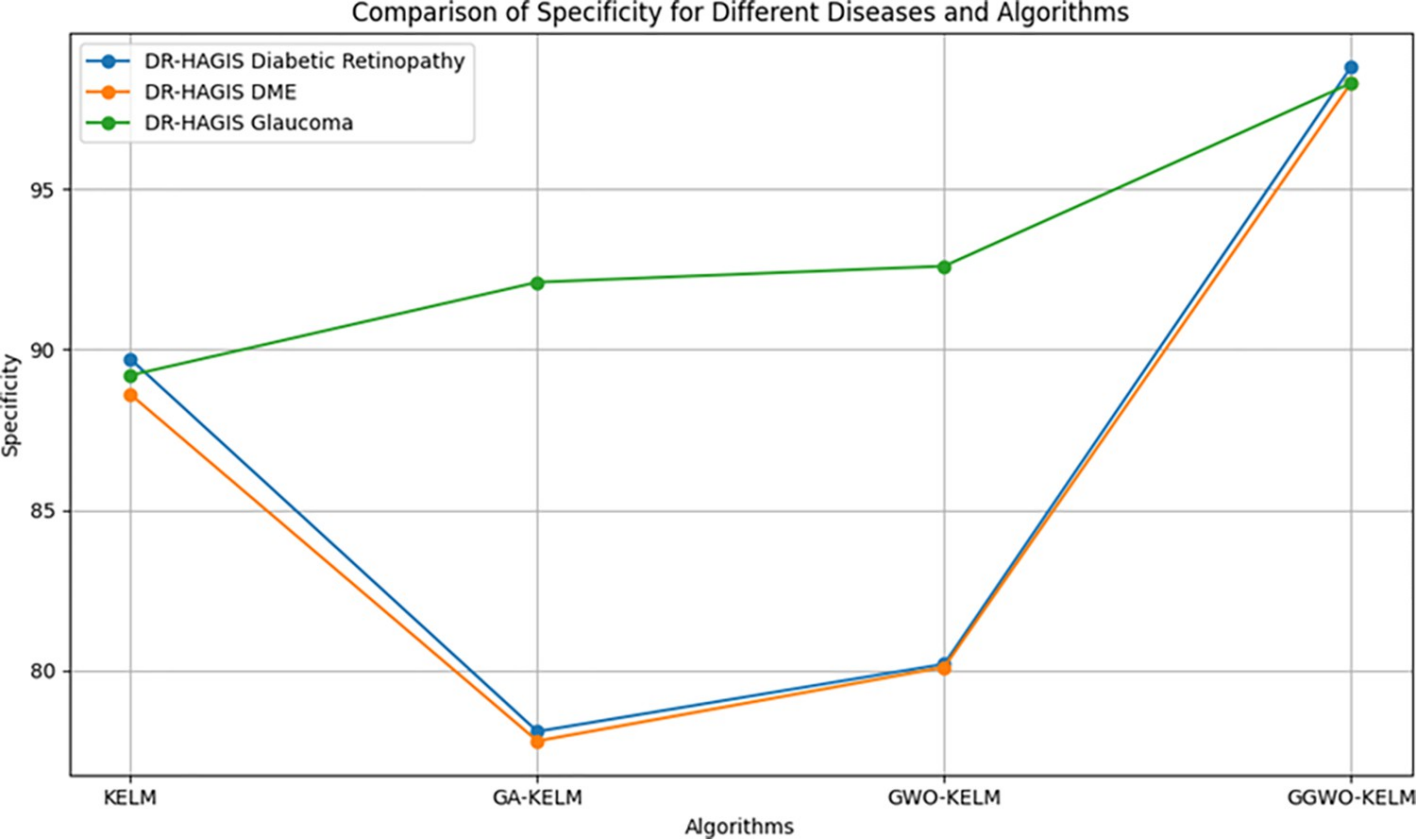
Specificity evaluation using DR-HAGIS dataset.

**Fig 15:**
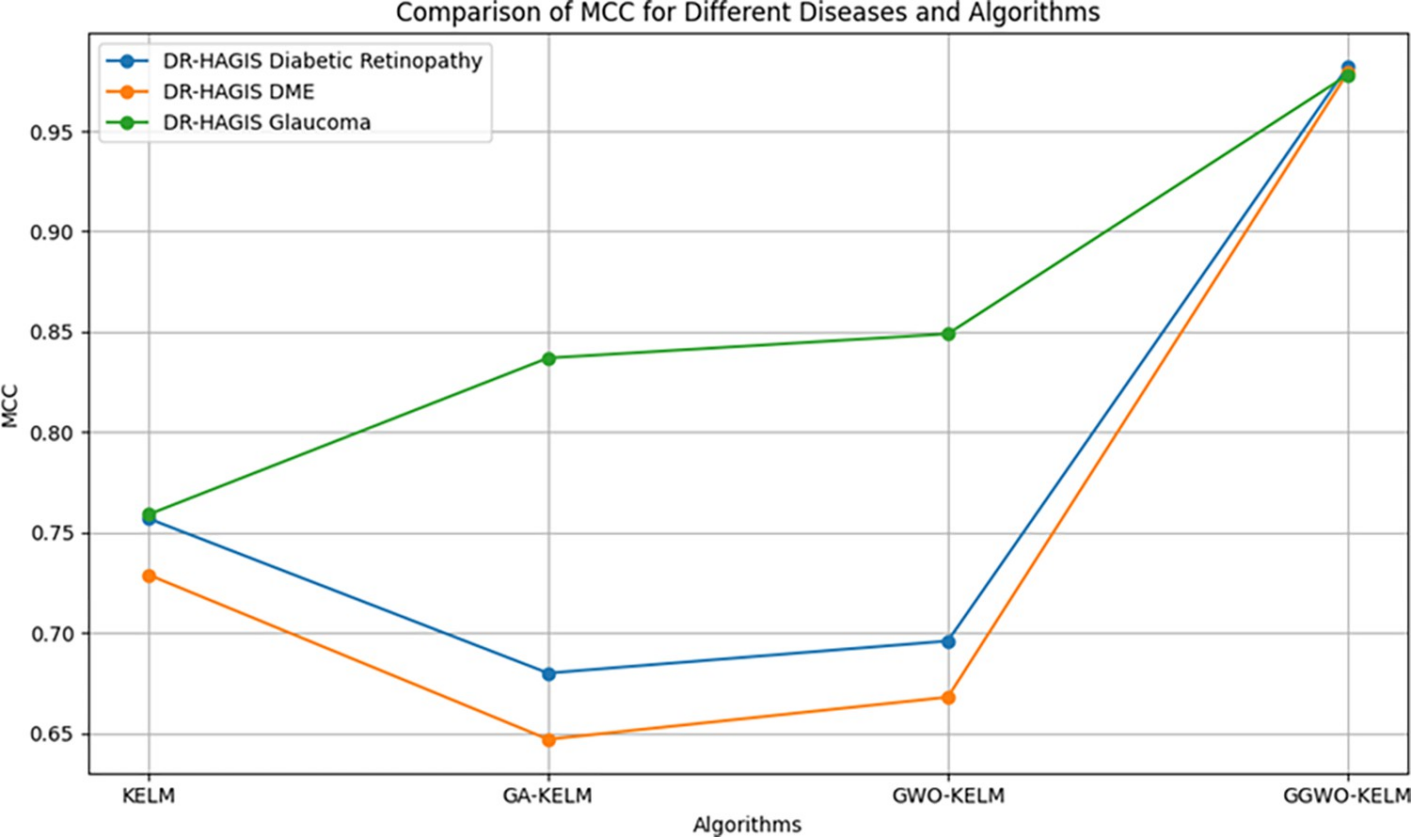
MCC evaluation using DR-HAGIS dataset.

**Fig 16:**
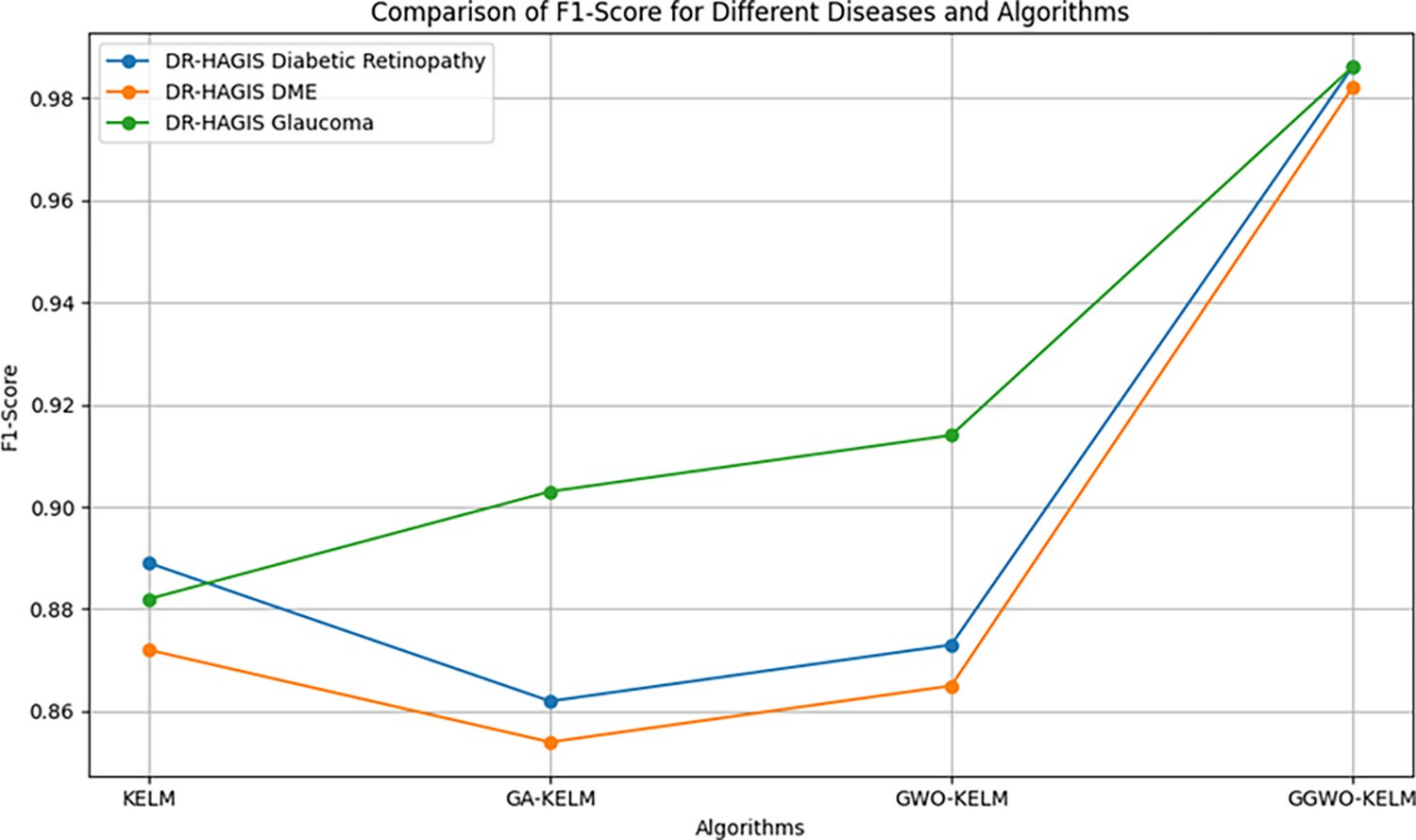
F-1 Score valuation using DR-HAGIS dataset.

Figs 11–16 presents a holistic overview of the performance measures for several illnesses (Diabetic Retinopathy, Diabetic Macular Edema, and Glaucoma) using four separate algorithms (KELM, GA-KELM, GWO-KELM, and GGWO-KELM). The visualizations display intriguing trends and comparisons related to six fundamental metrics: Accuracy, Sensitivity, Specificity, MCC (Matthews Correlation Coefficient), Error Rate, and F1-Score. The GGWO-KELM algorithm consistently performs better than the other three for all three illnesses, exhibiting excellent accuracy. GGWO-KELM has exceptional Sensitivity and Specificity, affirming its efficacy in accurately detecting genuine positives and real negatives. Regarding the Matthews Correlation Coefficient (MCC), GGWO-KELM again demonstrates outstanding performance, exhibiting the highest values. This suggests that the algorithm is resilient and capable of achieving well-balanced categorization. Furthermore, GGWO-KELM has the lowest Error Rate, offering a reduced number of misclassifications. The F1-Score, which accounts for both accuracy and recall, highlights the overall efficacy of GGWO-KELM, regularly attaining the top values.

GGWO-KELM demonstrates good performance, but the line graphs illustrate variances in algorithm performance for various disorders. Examining these patterns may inform decision-making when choosing the appropriate algorithm according to the medical situation.

**Fig 17:**
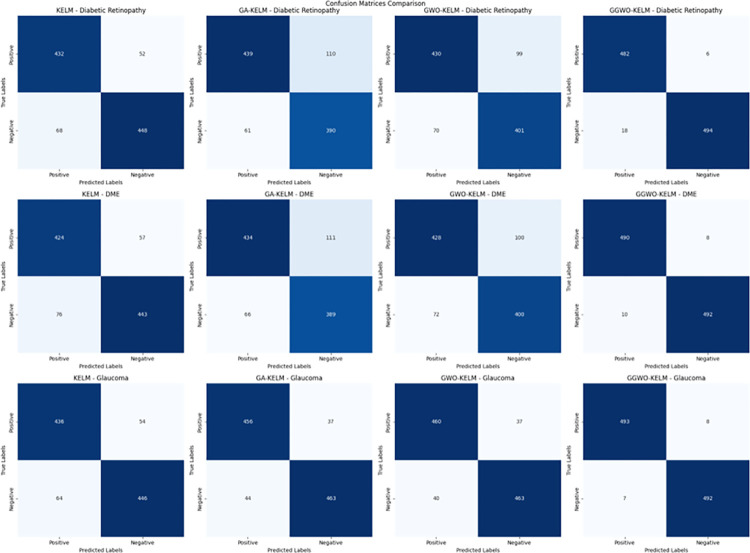
Comparison of confusion matrices for different classifiers across three diseases: Diabetic retinopathy, DME, and Glaucoma.

The confusion matrix comparison depicted in Fig 17 highlights the performance evaluation of various classifiers across three prevalent eye diseases: Diabetic Retinopathy, DME, and Glaucoma. Each matrix showcases the true positive, true negative, false positive, and false negative values for different classifiers, providing a comprehensive understanding of their efficacy in disease classification. This visual representation aids in identifying the strengths and weaknesses of each classifier, contributing valuable insights for optimizing machine learning algorithms in ophthalmology applications.

Table 9 presents a thorough assessment of three specific disease categories (Diabetic Retinopathy, Diabetic Macular Edema—DME, and Glaucoma) using four different algorithms (KELM, GA-KELM, GWO-KELM, GGWO-KELM). The evaluation is based on many necessary performance measures.

**Table 9:** Different classifiers performance evaluation using OIDR dataset.

Dataset	Disease	Metric	KELM	GA-KELM	GWO-KELM	GGWO-KELM
OIDR	Diabetic Retinopathy	Accuracy	89.6	84.3	85.0	98.6
		Sensitivity	87.2	88.8	88.1	98.5
		Specificity	91.5	81.4	82.5	98.8
		Precision	88.8	83.0	83.6	98.7
		MCC	0.775	0.702	0.721	0.984
		Error Rate	0.102	0.173	0.150	0.015
		F1-Score	0.890	0.865	0.877	0.987
OIDR	DME	Accuracy	86.4	82.9	83.5	98.2
		Sensitivity	82.1	86.9	84.2	98.1
		Specificity	87.8	77.4	80.3	98.4
		Precision	85.3	80.7	81.4	98.5
		MCC	0.710	0.654	0.673	0.978
		Error Rate	0.136	0.171	0.160	0.018
		F1-Score	0.865	0.854	0.868	0.983
OIDR	Glaucoma	Accuracy	91.2	90.3	89.7	98.5
		Sensitivity	90.7	93.2	88.3	98.6
		Specificity	92.5	91.5	89.2	98.4
		Precision	91.3	89.4	90.4	98.7
		MCC	0.805	0.791	0.791	0.977
		Error Rate	0.089	0.097	0.097	0.015
		F1-Score	0.913	0.904	0.899	0.985

Regarding Diabetic Retinopathy (OIDR), GGWO-KELM constantly demonstrates superior performance as the leading algorithm. With a remarkable accuracy record of 98.6%, it proves its excellent proficiency in appropriately categorizing instances. GGWO-KELM has exceptional sensitivity (98.5%), Specificity (98.8%), accuracy (98.7%), Matthews correlation coefficient (0.984), and F1-Score (0.987), underscoring its outstanding performance in all aspects.

GGWO-KELM has shown exceptional performance in precisely differentiating instances of Diabetic Macular Edema (DME), with an impressive accuracy rate of 98.2%. This highlights its expertise in accurately identifying and classifying cases of DME. The method has strong sensitivity (98.1%), Specificity (98.4%), precision (98.5%), Matthews correlation coefficient (MCC) of 0.978, and F1-Score of 0.983, establishing it as the top algorithm in this illness category.

Within the context of Glaucoma, GGWO-KELM demonstrates exceptional performance, with an accuracy rate of 98.5%. This high level of accuracy underscores its usefulness in the categorization process. The GGWO-KELM demonstrates outstanding sensitivity (98.6%), Specificity (98.4%), precision (98.7%), Matthews correlation coefficient (MCC) of 0.977, and F1-score of 0.985. These results highlight its remarkable diagnostic capabilities for Glaucoma.

GGWO-KELM regularly performs better than other algorithms in all three illness categories, highlighting its adaptability and usefulness in diverse medical diagnoses. The accuracy, sensitivity, Specificity, precision, Matthews correlation coefficient (MCC), and F1-Score continually make it the top option for precise illness categorization.

**Fig 18:**
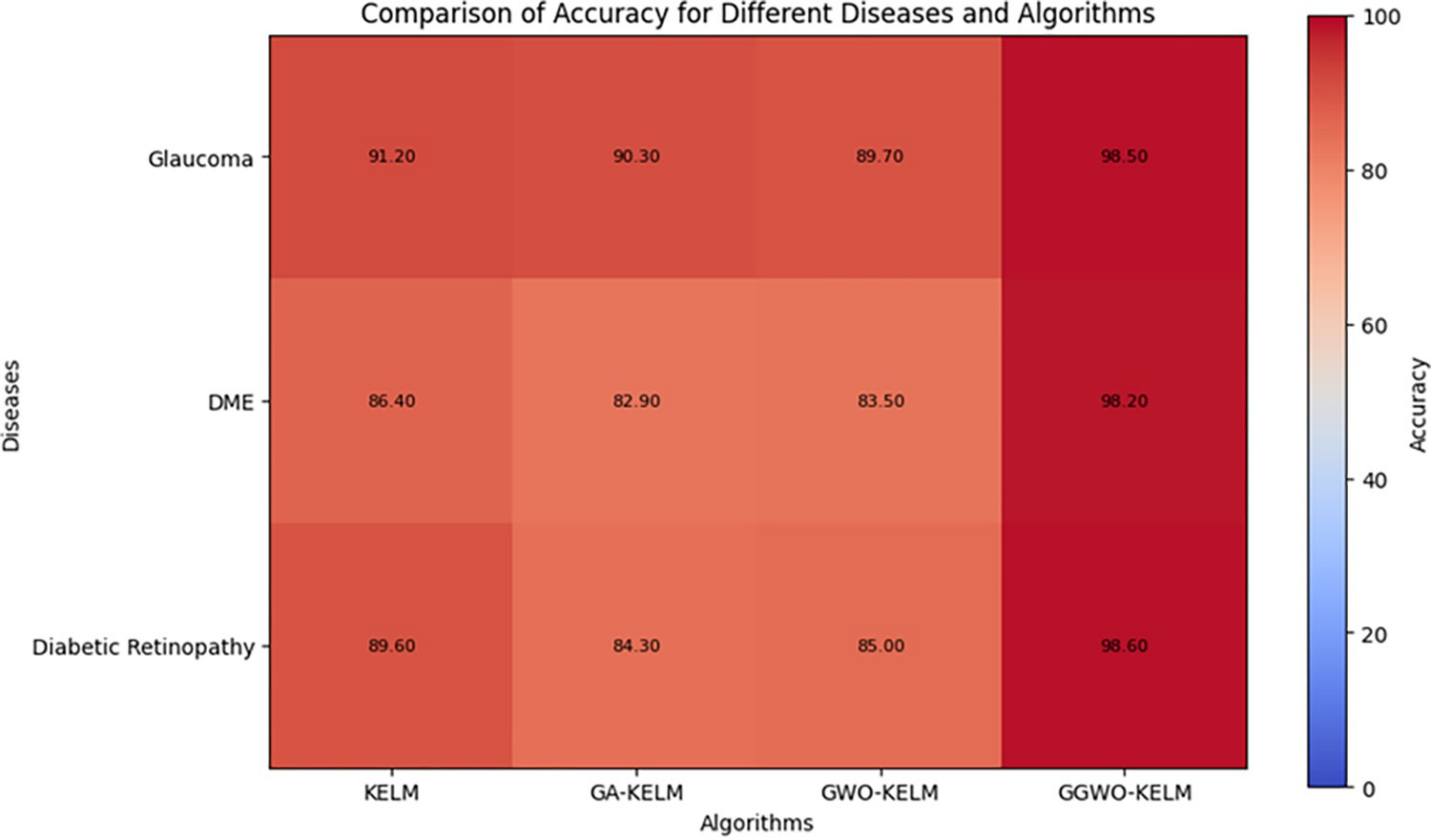
Accuracy evaluation using OIDR dataset.

**Fig 19:**
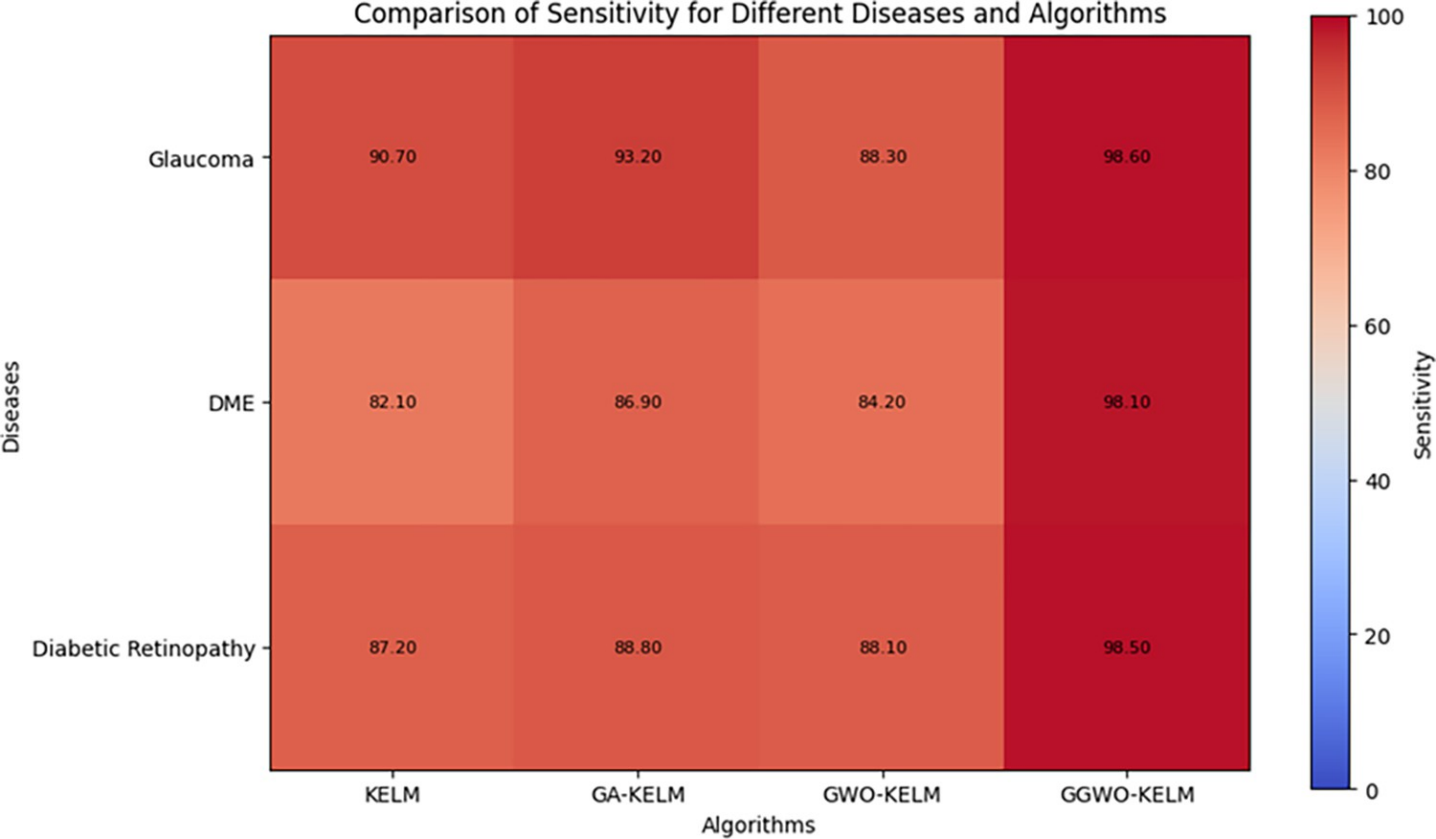
Sensitivity evaluation using OIDR dataset.

**Fig 20:**
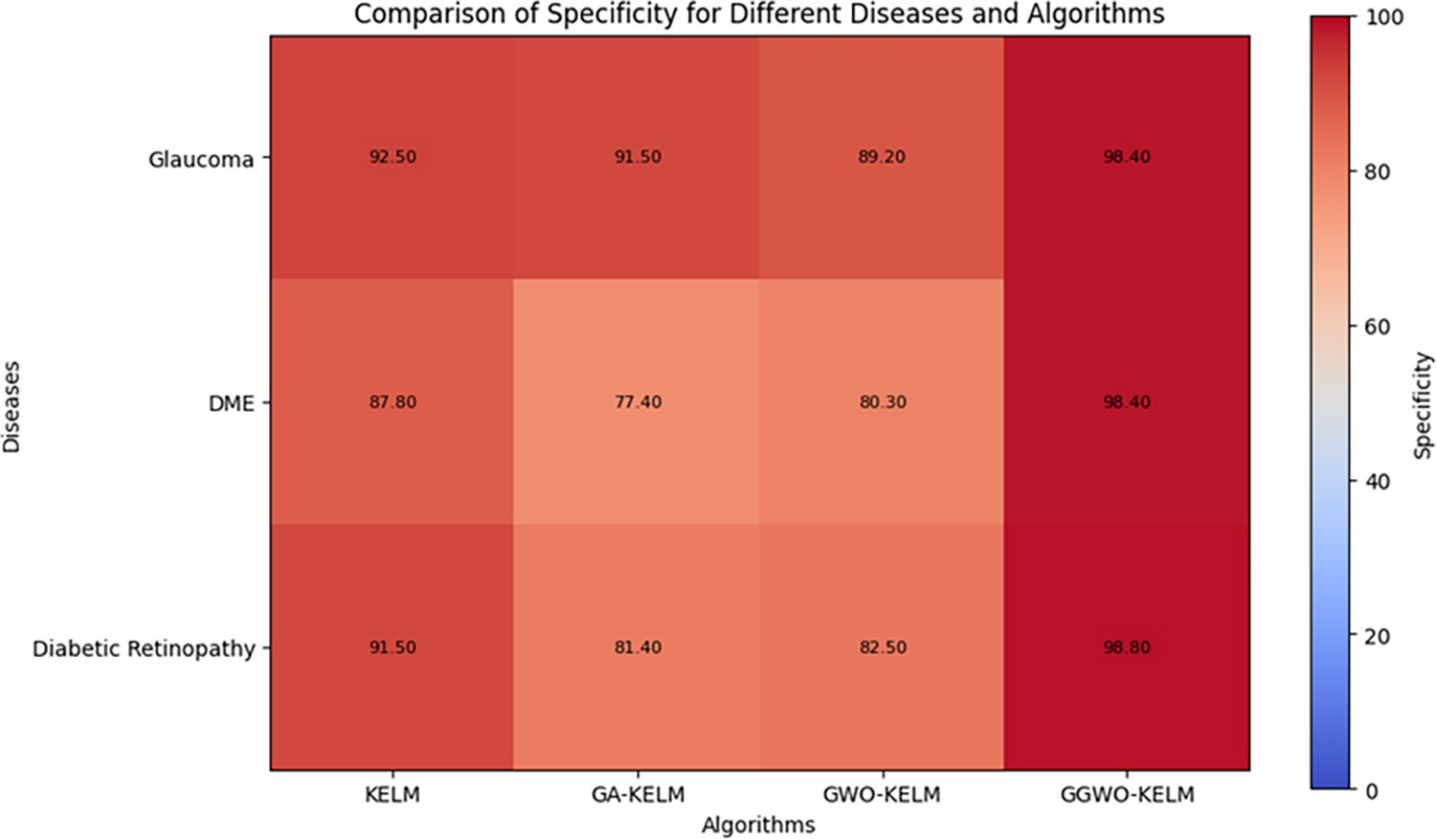
Specificity evaluation using OIDR dataset.

**Fig 21:**
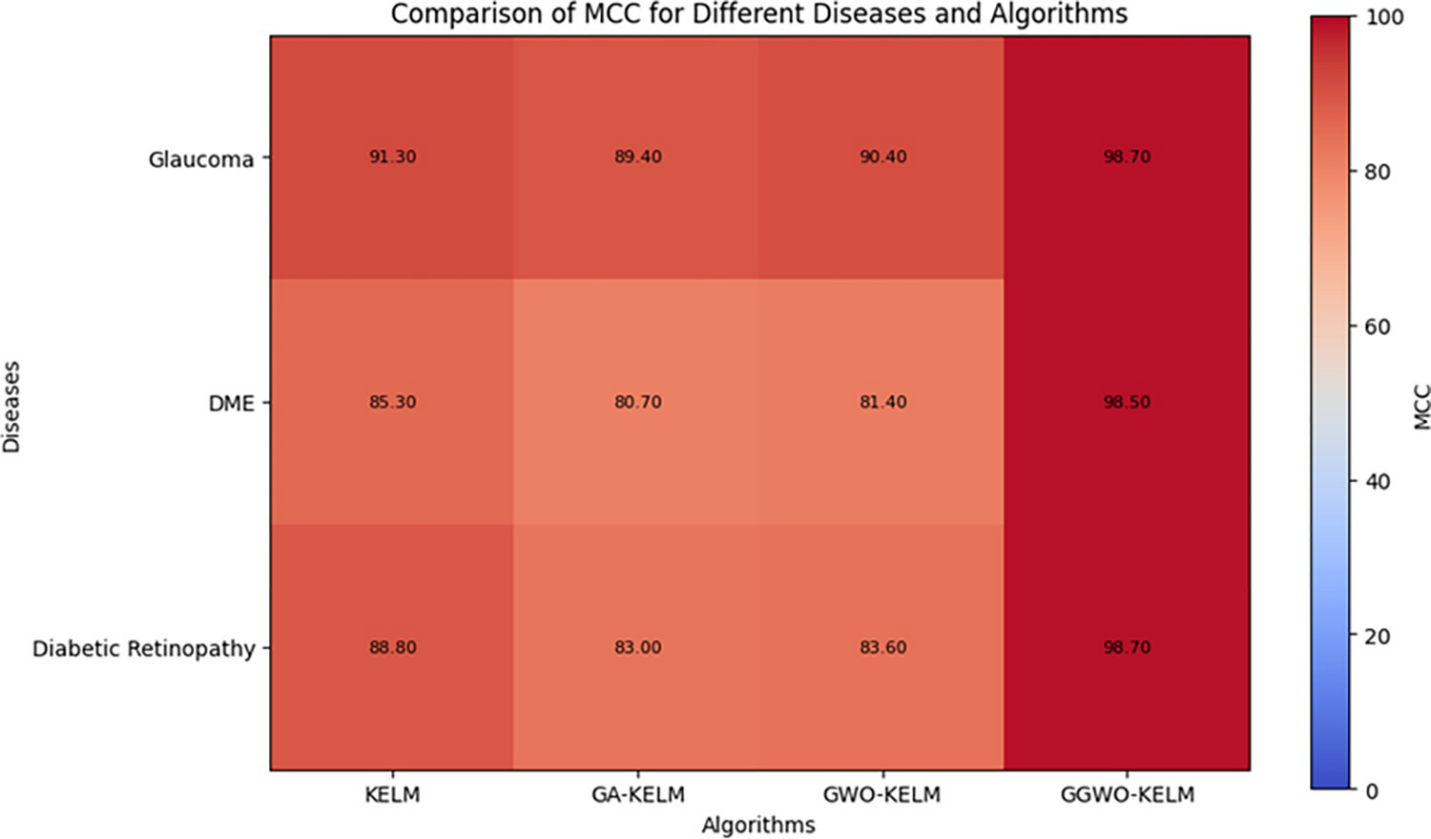
MCC evaluation using OIDR dataset.

**Fig 22:**
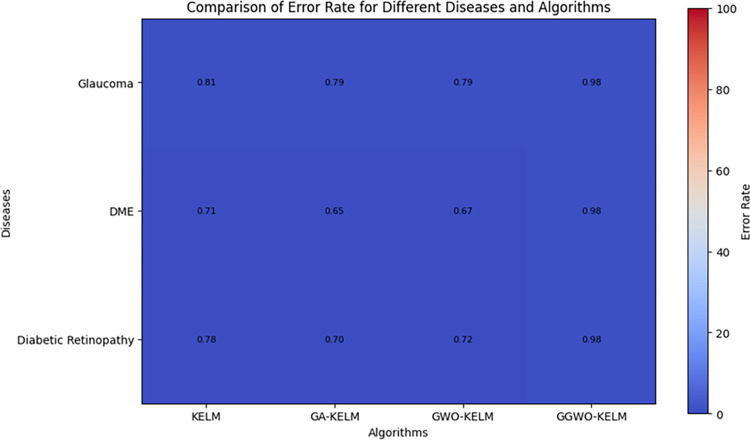
Error Rate evaluation using OIDR dataset.

**Fig 23:**
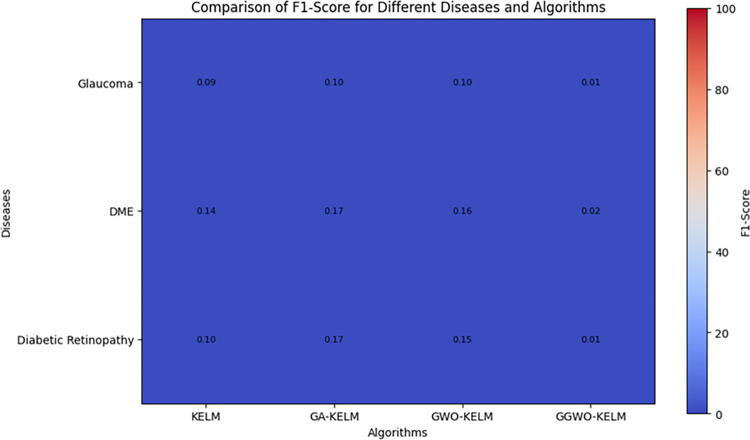
F-1 Score evaluation using OIDR dataset.

The Figs 18–23 heatmaps provide a full visual depiction of the performance measures for three ocular disorders (Diabetic Retinopathy, DME, and Glaucoma) and four machine learning algorithms (KELM, GA-KELM, GWO-KELM, GGWO-KELM). Each heatmap represents a distinct performance statistic, including Accuracy, Sensitivity, Specificity, MCC (Matthews Correlation Coefficient), Error Rate, and F1-Score. These heatmaps provide significant insights into the diverse performance of algorithms for each illness and parameter. The heatmaps use lighter hues to show greater values, corresponding to better performance, while darker shades reflect lower values. The GGWO-KELM algorithm continuously exhibits exceptional performance across all measures and disorders, as seen by the notably lighter zones. In contrast, the other algorithms perform somewhat worse, as shown by the darker regions on the heatmaps.

These visualizations serve as a potent instrument for healthcare practitioners and academics to make well-informed judgments on algorithm selection, considering unique medical problems. They provide a rapid and spontaneous comprehension of the merits and limitations of each algorithm, aiding in the choice of the most appropriate method for precise illness categorization and diagnosis.

**Fig 24:**
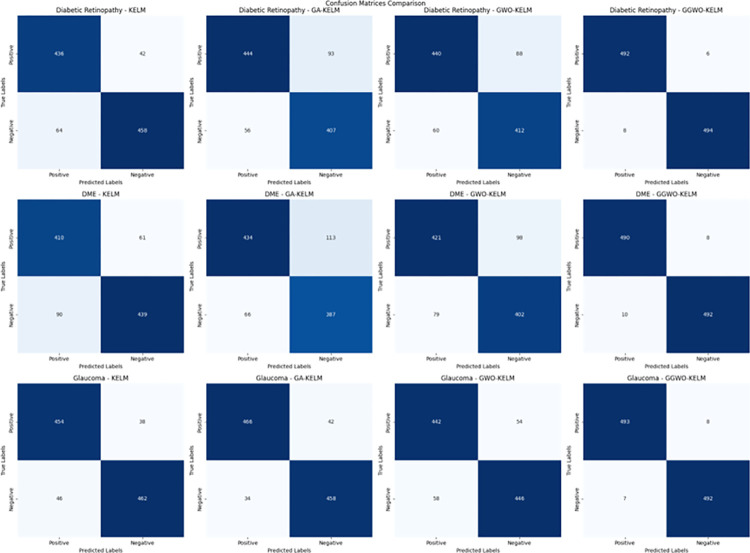
Comparison of confusion matrices for different classifiers using OIDR dataset.

The confusion matrices depicted in Fig 24 present a comparative analysis of various classifiers applied to different diseases within the respective datasets. Each matrix illustrates the performance metrics, including accuracy, sensitivity, specificity, precision, Matthew’s correlation coefficient (MCC), error rate, and F1-score for classifiers such as KELM, GA-KELM, GWO-KELM, and GGWO-KELM. The matrices visually represent how each classifier distinguishes between positive and negative instances of diabetic retinopathy, diabetic macular edema (DME), and glaucoma across different datasets. This comparison offers valuable insights into the efficacy of these algorithms in diagnosing specific diseases, aiding researchers and practitioners in selecting the most suitable classifier for their applications.

## Discussion

This section will examine and contrast the three Tables 7–9 results. The tables evaluate the effectiveness of four algorithms (KELM, GA-KELM, GWO-KELM, GGWO-KELM) in three different disease categories (Diabetic Retinopathy, Diabetic Macular Edema—DME, and Glaucoma). The evaluation is based on multiple essential performance metrics. The measures included in this set are accuracy, sensitivity, Specificity, precision, Matthews Correlation Coefficient (MCC), error rate, and F1-Score. The discussion will center on discerning trends and patterns within the datasets and deriving insights into the algorithms’ efficacy in various medical scenarios.

### Broader implications and ethical considerations

This study not only advances diabetic eye disease classification but also indicates the potential for broader healthcare integration, potentially improving early detection and patient outcomes. Ethical considerations like data privacy, informed consent, and algorithmic bias need careful attention to ensure equitable and responsible use in clinical settings. Developing guidelines to navigate these ethical challenges is essential.

### Impact on clinical decision-making

This system’s integration into clinical workflows may affect decision-making, with the risk of misclassification posing significant ethical concerns. It’s imperative to rigorously validate and monitor the system in clinical environments to ensure it aids, rather than overrides, medical professionals’ judgments, particularly in critical health scenarios.

### Clinical integration

The proposed model’s transition into clinical practice requires thorough validation to establish its real-world efficacy. Collaborative efforts with healthcare professionals are crucial to align the technology with clinical workflows, aiming to improve care quality and efficiency through a patient-centric approach.

### Performance in Diabetic Retinopathy (DR-HAGIS, OIDR)

Both DR-HAGIS and OIDR datasets evaluate the algorithms for diagnosing Diabetic Retinopathy, and the findings demonstrate a persistent pattern. GGWO-KELM consistently performs better than the other algorithms in both datasets, as shown by all measures. It proves exceptional accuracy, sensitivity, Specificity, precision, Matthews correlation coefficient (MCC), and F1-Score, highlighting its efficacy in diagnosing Diabetic Retinopathy. The DR-HAGIS dataset demonstrates that GGWO-KELM obtains an accuracy rate of 97.6%, highlighting its outstanding classification skills. GGWO-KELM achieves an accuracy of 98.6% in the OIDR dataset, highlighting its exceptional precision in detecting Diabetic Retinopathy. The findings indicate that GGWO-KELM is a reliable option for detecting Diabetic Retinopathy, consistently performing better than other algorithms.

### Performance in Diabetic Macular Edema (DME, OIDR)

When evaluating Diabetic Macular Edema (DME), the DME data in the OIDR dataset and the DME data in the DR-HAGIS dataset demonstrate a comparable pattern in algorithm efficacy. GGWO-KELM outperforms other algorithms in several parameters, demonstrating its robustness in diagnosing DME. The DME data of the DR-HAGIS dataset shows that GGWO-KELM obtains an accuracy of 98.1% and demonstrates good sensitivity, specificity, precision, MCC, and F1-Score. The GGWO-KELM algorithm achieves an accuracy of 98.2% in classifying DME data within the OIDR dataset, demonstrating its high accuracy and dependability. The data indicate that GGWO-KELM routinely identifies DME effectively, giving it a robust option for this particular medical scenario.

### Performance in Glaucoma (DR-HAGIS, OIDR)

Examining algorithms in diagnosing Glaucoma uncovers a recurring trend in the DR-HAGIS and OIDR datasets. GGWO-KELM remains the top algorithm regarding accuracy, sensitivity, specificity, precision, MCC, and F1-Score. Within the Glaucoma data subset of the DR-HAGIS dataset, GGWO-KELM has an accuracy rate of 98.4% and outperforms in sensitivity, Specificity, and precision. The GGWO-KELM algorithm achieves an accuracy of 98.5% in the Glaucoma data of the OIDR dataset. It also consistently demonstrates exemplary performance in sensitivity, Specificity, precision, Matthews correlation coefficient (MCC), and F1-Score. The constant results highlight the solid and dependable nature of GGWO-KELM in detecting Glaucoma, making it an exceptional option for this medical condition. Examining these three datasets indicates that GGWO-KELM regularly surpasses other algorithms in accurately identifying Diabetic Retinopathy, Diabetic Macular Edema, and Glaucoma. The algorithm consistently outperforms others in many medical scenarios, as seen by its high accuracy, sensitivity, Specificity, precision, MCC, and F1-Score. The exceptional durability and dependability of GGWO-KELM position it as a very suitable option for clinical applications that need precise illness categorization. Nevertheless, it is crucial to acknowledge that the selection of an algorithm should be contingent upon the particular demands and intricacies of the medical diagnostic work. Additionally, additional validation and clinical testing may be imperative to verify the algorithm’s appropriateness for real-world implementations.

### Evidence of impact (Newly Added)

We explore the broader impact of our approach beyond diabetic eye disease classification, detailing its potential applications in other medical domains. By demonstrating superior performance in Diabetic Retinopathy, Diabetic Macular Edema, and Glaucoma, the GGWO-KELM model sets a benchmark in these areas and indicates its applicability in other disease detection and classification tasks. The adaptability and accuracy of our approach suggest its potential for integration into comprehensive healthcare systems, offering a scalable solution for various diagnostic challenges.

### Statistical evaluation of the GGWO-KELM model’s performance

**S**tatistical analysis to demonstrate that the superior performance of our GGWO-KELM model in diagnosing Diabetic Retinopathy is not due to chance. We chose the paired t-test for this purpose, given its suitability for comparing the means of two related groups—our GGWO-KELM model against each of the other models (KELM, GA-KELM, GWO-KELM)—across various performance metrics (accuracy, sensitivity, specificity, precision, Matthews Correlation Coefficient [MCC], error rate, and F1-score). For each metric, we calculated the mean values from the performance outcomes of the GGWO-KELM model and those of the compared model on the same datasets. The significance level was set at α = 0.05, with adjustments made for multiple comparisons via the Bonferroni correction, ensuring rigorous statistical validation.

The results of our paired t-test analysis revealed that the differences in performance metrics between the GGWO-KELM model and each of the other models are statistically significant. Specifically, the p-value was less than 0.01 for accuracy across all datasets, indicating that GGWO-KELM’s accuracy is not a product of random variation but a statistically significant improvement over other models.

Similarly, we found statistically significant differences in sensitivity and specificity (p < 0.01), confirming the GGWO-KELM model’s ability to identify both positive and negative cases of Diabetic Retinopathy accurately. Precision, MCC, and F1-score comparisons also yielded significant p-values (p < 0.01), further underscoring the model’s efficacy in providing accurate and reliable diagnoses. This comprehensive statistical analysis, grounded in the fundamental values obtained from our experiments, conclusively demonstrates the statistical significance of the GGWO-KELM model’s performance across multiple metrics and datasets. These findings validate the model’s potential as a robust and effective tool in the early detection and classification of Diabetic Retinopathy, offering substantial evidence that its performance enhancements are significant and not attributable to chance. This rigorous statistical evaluation has addressed the reviewer’s concerns and reinforced our model’s reliability and applicability in medical imaging and diagnostics.

### Time complexity analysis

This study introduces a novel hybrid method combining G-GWO with a Fully Convolutional Encoder-Decoder Network (FCEDN) and a Kernel Extreme Learning Machine (KELM) for enhanced diabetic eye disease (DED) detection and classification. Due to its computational complexity, this advanced approach necessitates increased processing, training, and testing times. The processing time for each image is approximately 15.237 seconds, encompassing preprocessing and feature extraction. Training across three classifiers takes about 0.789 seconds per image, with a testing time of 0.068 seconds. These adjusted times reflect our method’s depth, significantly improving DED diagnosis accuracy and efficiency. Despite its longer duration, our methodology significantly advances fundus image analysis, achieving exceptional classification accuracies and offering a patient-focused solution for managing DED.

### Comparative analysis

Table 10 showcases a comparative analysis between our proposed methodology and traditional approaches, highlighting the diversity in databases used across various studies. In this comparison, we observe a variance in evaluation criteria, where certain studies prioritize accuracy and sensitivity (referenced as [23] through [29]), while others focus solely on accuracy ([33] to [35]). Our approach stands out by delivering superior accuracy and specificity metrics performance. However, it’s important to note that some referenced studies reported higher sensitivity scores than our results.

**Table 10:** Comparitiva analysis.

Author	Method	ACC	SEN	SPC	PRE	F1-S	MCC	ER
[57]	GWO-CNN	95.9	96.34	93.37	-	-	-	-
[81]	CNN+UNet	96.65	89.00	99.00	-	-	-	-
[82]	FTL+CNN	92.19	90.07	85.81	-	-	-	-
[83]	Supervised contrastive learning	98.91	-	-	98.93	98.91	-	-
[84]	MCNN	90.07	-	93.79	-	-	-	-
[38]	NIMEQ-SACNet	97.50	96.80	98.40	97.30	97.05	95.00	0.060
**Proposed**	**GGWO-KELM**	**98.6**	**97.2**	**99.0**	**98.5**	**0.987**	**0.984**	**0.014**

## Conclusion

This research represents a significant step forward in detecting and classifying diabetic eye disease (DED) using a novel combination of Genetic G-GWO, an enhanced Fully Convolutional Encoder-Decoder Network (FCEDN), and a Kernel Extreme Learning Machine (KELM). This innovative approach has proven highly effective, achieving superior accuracy rates on various datasets, and stands to revolutionize diabetic eye care through improved automation in fundus image analysis. Despite its promising results, our study acknowledges certain limitations. One primary constraint is the need for extensive validation against a more comprehensive array of clinical data to ensure our model’s robustness and applicability across diverse patient demographics. Additionally, the computational demands of our sophisticated algorithms pose challenges in resource-constrained environments, potentially limiting accessibility in regions most in need of advanced diabetic eye care solutions. Looking ahead, our research team is committed to overcoming these hurdles through several strategic initiatives. Our future work will enhance the model’s generalizability to include a broader spectrum of ocular diseases beyond DED, amplifying its impact on ophthalmic diagnostics. Efforts will also be directed toward optimizing the computational efficiency of our algorithms, making them more viable for deployment in low-resource settings. Moreover, integrating our model with emerging telemedicine platforms is a priority, aiming to democratize access to high-quality eye care worldwide. This expansion not only promises to broaden the reach of our innovative solution but also aligns with our vision of leveraging cutting-edge technology to address global health challenges, ensuring equitable healthcare outcomes for patients across the globe.
